# Detumescence Analgesic Plaster mitigates knee osteoarthritis via active ingredients targeting mitochondrial complex 1/AMPK/MYL3-regulated cartilage homeostasis

**DOI:** 10.1186/s13020-025-01215-w

**Published:** 2025-10-20

**Authors:** Chunxia Li, Weijie Li, Yue Yin, Xiaomei Xiang, Lu Fu, Ping Wang, Yanqiong Zhang, Haiyu Xu

**Affiliations:** 1 State Key Laboratory for Quality Ensurance and Sustainable Use of Dao-Di Herbs, Institute of Chinese Materia Medica, China Academy of Chinese Medical Sciences, Beijing, China; 2 College of Pharmacy, Liaoning University of Traditional Chinese Medicine, Liaoning, China

**Keywords:** Detumescence Analgesic Plaster, Knee osteoarthritis, Mitochondrial complex 1, Cartilage degeneration, Mitochondrial dysfunction

## Abstract

**Background:**

The Detumescence Analgesic Plaster (DAP) has been widely used in clinical practice for knee osteoarthritis (KOA) treatment, yet its active ingredients and molecular mechanisms remain incompletely understood.

**Purpose:**

This study aimed to systematically characterize DAP’s chemical composition and decipher its chondroprotective pathways in KOA.

**Methods:**

A papain-induced KOA rat model was employed to evaluate DAP’s therapeutic effects through behavioral assessments (mechanical withdrawal threshold, gait analysis) and histological evaluations (H&E, safranin O-fast green staining). UPLC-Q-TOF/MS combined with Franz diffusion cells identified DAP’s chemical profile. RNA-seq was performed to compare gene expression between KOA and DAP-treated groups, followed by protein–protein interaction (PPI) and gene co-expression network analysis to prioritize key targets. Validation was conducted using Western blot, qPCR, and immunohistochemistry. IL-1β-stimulated chondrocytes were used to screen active ingredients and validate their effects on mitochondrial function.

**Results:**

DAP treatment significantly alleviated pain, restored joint mobility, and preserved cartilage integrity in KOA rats. Chemical profiling identified 92 compounds, including 28 active ingredients with high transdermal permeability. RNA-seq revealed 206 DAP-reversed genes primarily associated with mitochondrial dysfunction, oxidative stress, and inflammatory signaling. Network analysis pinpointed 23 core targets, with mitochondrial complex I subunits (NDUFA5, NDUFA6, NDUFS6), AMPK, and MYL3 emerging as critical nodes in oxidative phosphorylation. DAP restored the expression of these targets in KOA cartilage. In vitro experiments demonstrated that 1,5-dicaffeoylquinic acid, verproside, and catalposide attenuated ROS production, enhanced ATP synthesis, and stabilized mitochondrial membrane potential via the NDUFA6/AMPK/MYL3 axis, thereby inhibiting chondrocyte apoptosis.

**Conclusion:**

This study provides the first evidence that DAP exerts chondroprotective effects by ameliorating mitochondrial dysfunction and oxidative stress in KOA through the mitochondrial complex I/AMPK/MYL3 signaling pathway. These findings offer a mechanistic basis for DAP’s clinical efficacy and highlight potential therapeutic targets for KOA management.

**Graphical Abstract:**

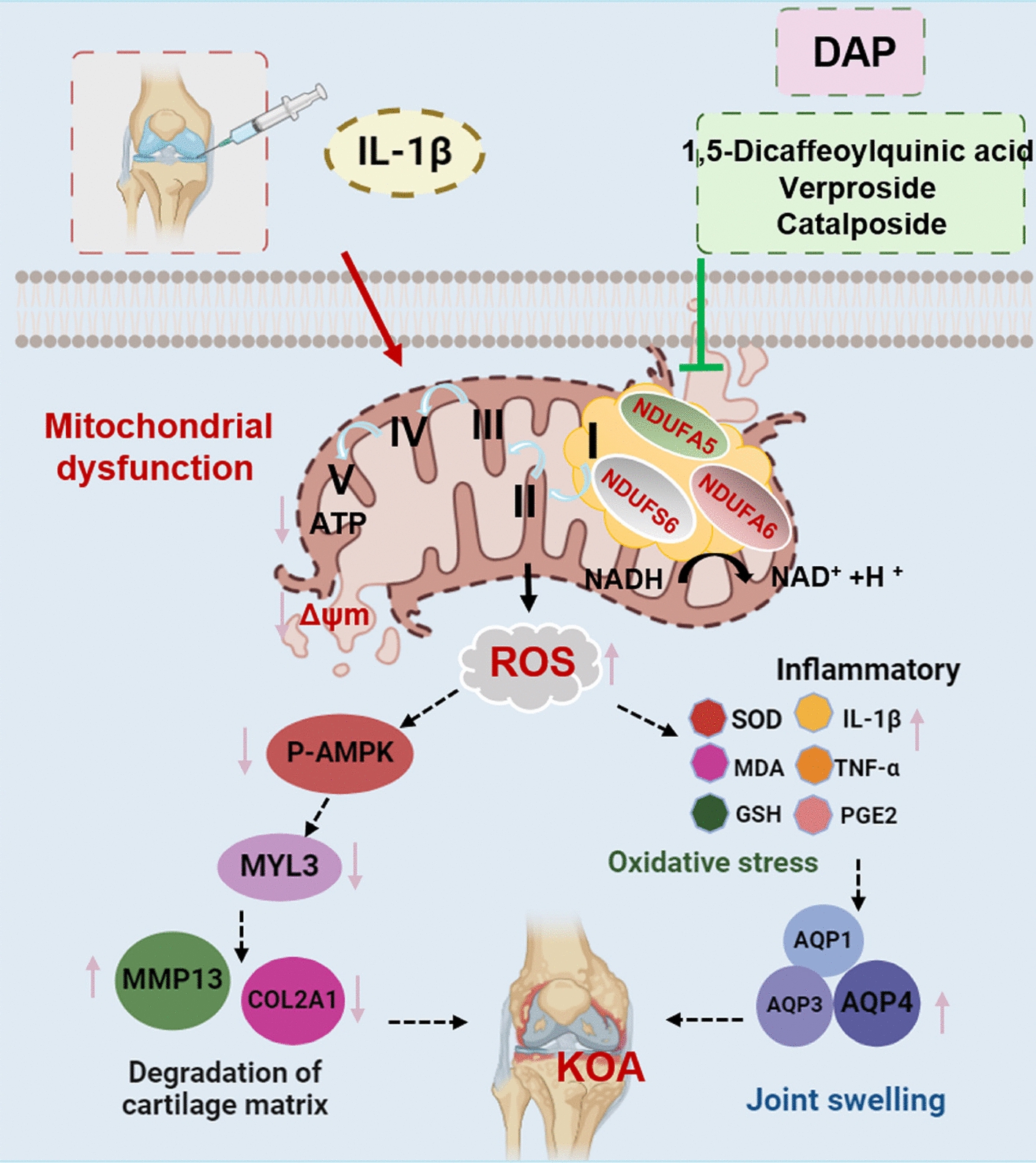

**Supplementary Information:**

The online version contains supplementary material available at 10.1186/s13020-025-01215-w.

## Introduction

Globally, osteoarthritis (OA) represents the predominant articular pathology, impacting nearly 250 million individuals and creating substantial healthcare challenges internationally [1]. Among the geriatric and middle-aged demographics, knee osteoarthritis (KOA) emerges as a frequently occurring degenerative condition affecting articular structures [2, 3]. KOA develops due to an imbalance in the production and breakdown of articular cartilage, extracellular matrix (ECM), and subchondral bone, coupled with inflammation and fibrosis of the synovium. The hallmark characteristic of this pathology involves articular cartilage's gradual deterioration, characterized by cellular dysfunction, matrix disintegration, architectural compromise, and ultimately complete functional failure of cartilaginous tissue in severe cases [4, 5]. Contemporary management approaches predominantly emphasize anti-inflammatory interventions and pain control modalities, providing temporary symptomatic relief without effectively targeting foundational pathophysiological mechanisms [6]. Therefore, there is an urgent need for effective clinical treatments to prevent cartilage degeneration during the progression of KOA [7].

Accumulated evidence indicates a strong correlation between mitochondrial dysfunction and the pathogenesis of KOA [8]. Mitochondria are vital for oxidative phosphorylation (OXPHOS) and adenosine triphosphate (ATP) production through respiratory complexes I to IV and ATP synthase (complex V) [9]. Chondrocytes affected by KOA exhibit compromised mitochondrial functionality, characterized by attenuated activities within respiratory complexes I through III, diminished ATP synthesis capacity, impaired mitochondrial membrane potential (MMP) integrity, and elevated markers of oxidative stress [10]. These alterations disrupt cellular homeostasis and can trigger the release of inflammatory mediators, leading to cartilage degeneration [11–13].

The initial component of the respiratory machinery, mitochondrial complex I, serves as a critical gateway for introducing electrons into the electron transport chain (ETC), thereby facilitating subsequent ATP synthesis processes [14]. In KOA pathology, cartilaginous tissue degradation occurs partially through mechanisms involving the aberrant accumulation of reactive oxygen species (ROS) and resultant oxidative cellular damage [15]. Prolonged impairment of respiratory chain functionality within mitochondria leads to reduced activity of adenosine 5'-monophosphate (AMP)-activated protein kinase (AMPK), consequently disrupting downstream signaling cascades [16]. Elevated levels of ROS can inhibit the AMPK pathway, further disrupting energy metabolism. AMPK is essential for maintaining mitochondrial homeostasis [17], and its activation in chondrocytes reduces oxidative stress, alleviates inflammation, and enhances cell viability. Conversely, decreased AMPK activity is associated with cartilage degeneration and the onset of KOA [18–20]. Therefore, therapeutic interventions aimed at restoring mitochondrial metabolic balance and counteracting oxidative damage mechanisms represent promising strategies for effective management of KOA.

Unlike conventional oral and injectable therapies, the Detumescence Analgesic Plaster (DAP), administered as a transdermal patch, has demonstrated clinical efficacy in treating KOA, particularly in alleviating joint swelling and pain [21]. Despite its clinical applications [22–24], a comprehensive understanding of both the active constituents and the mechanistic pathways through which DAP exerts its therapeutic effects on KOA remains incomplete. Our investigation thoroughly evaluated the therapeutic effects of DAP using a papain-induced KOA rat model and identified its transdermal active ingredients. To elucidate the underlying molecular pathways involved in DAP in alleviating joint edema, pain, and cartilage degeneration, we employed transcriptomic analysis through RNA sequencing methodologies. Molecular biology techniques were utilized to validate key genes and associated pathways within the network. Finally, the active ingredients in DAP were screened using an interleukin-1 beta (IL-1β)-induced cartilage degeneration model.

## Materials and methods

### Animals

Sprague Dawley (SD) rats (Specific Pathogen Free (SPF), male, 200–220 g) (animal experiments), 4-week-old male SD rats (cell isolation) were acquired from Vital River Laboratory Animal Technology Co.,Ltd, (SCXK,2021-0011, Beijing, China). The experimental animals were maintained in pathogen-controlled facilities under standardized environmental parameters (ambient temperature maintained at 23 ± 2 °C with relative humidity of 55 ± 5%), receiving unrestricted access to regular rodent diet and drinking water. The Institute of Chinese Materia Medica’s Experimental Animal Ethics Committee reviewed and granted approval for all protocols involving animal utilized in this research (Approval No: 2023B312).

### Drugs

The DAP (China Approved Drug Number B20020890), comprising seeds of *Artemisia halodendron* Turcz. ex Bess (AHT, Cat. No. 230107, 0.4 g/per patch), menthol (Cat. No.230504, purity: 98.60%, 0.0016 g/mL), and *Veronica linariifolia* Pall. ex Link subsp. Dilatate (Nakai et Kitagawa) Hong (VLP, Cat. No. 220401, 0.4 g/mL), was sourced from Shaanxi Momentum Qixuehe Pharmaceutical Co..Ltd (Shaanxi, China). Qizheng Xiaotong Plaster (QZXTP), procured from Tibet Cheezheng Tibetan Medicine Co., Ltd. (China Approved Drug Number Z54020113, Xizang, China), was used as a positive control. The preparation process involved combining 20 g of AHT, 0.8 g of VLP, and 0.04 g of menthol at a ratio of 50:1, transferring the greeze dried powder mixture into a 200 mL beaker. Subsequently, 100 mL of a 25% ethanol solution was added to the beaker to prepare a 100 mL raw material drug mixture (Additional file 1: Materials S1).

### Grouping, KOA modeling, and treatment

Rats were randomly allocated to six groups: (1) the control group (*n* = 12), (2) the KOA model group (*n* = 12), (3) the DAP low-dose (DAP-L, 0.0367 g/kg, 1.0-fold clinical dose, *n* = 12) group, (4) the DAP medium-dose (DAP-M, 0.0735 g/kg, 2.0-fold clinical dose, *n* = 12) group, (5) the DAP high-dose (DAP-H, 0.1470 g/kg, 4.0-fold clinical dose, *n* = 12) group, and (6) the QZXTP, (Positive, 0.1058 g/kg, 1.0-fold clinical dose, *n* = 8) group. The clinical equivalent dose for rats was 0.0367 g/kg, based on a body surface area conversion factor of 6.17 [25] (Additional file 2: Table S1).

KOA was induced using papain enzyme [26]. After anesthetizing rats with 2% pentobarbital sodium (Sigma Aldrich, USA), the rats were fixed, and hair was shaved around a 2 cm area of the right knee joint. Subsequent to antiseptic preparation with iodine solution, intra-articular administration of 0.1 mL solution containing freshly constituted 4% papain combined with L-cysteine (Solarbio, Beijing, China) was performed at the right knee articulation, followed by secondary antiseptic application. The experimental protocol involved administering modeling agent injections to all treatment cohorts on days 1, 4, and 7 of the experimental periods, while control group received vehicle solution only; subsequently, standard husbandry practices were maintained throughout the ensuing 5-week duration.

Therapeutic intervention with DAP commenced on the 8th day post-model establishment, with topical application encircling the affected right knee articulation continuing for a five -week treatment course. To maintain experimental integrity by preventing oral interaction with the medicinal application site and minimizing inter-subject contact that might compromise result validity, each animal was provided separate housing accommodations following administration of the therapeutic intervention (Additional file 1: Methods S2).

### Joint swelling degree

Observations were conducted on knee joint inflammation in the experimental animal model. Following model establishment, measurements of the right knee joint diameter at consistent anatomical locations were performed at 4-day intervals utilizing a vernier caliper manufactured by Green Forest (Yantai, China).

### Mechanical withdrawal threshold

Pain sensitivity measurements utilized a calibrated von Frey device (manufactured by IITC Life Science, California, USA) to determine tactile response thresholds. Assessment procedures adhered to our research group's established methodology, and final MWT values represented an average calculated from three independent measurements [27].

### Load difference test

Three days prior to sampling, rats were trained and tested for weight differences. Each rat was placed in a slightly tilted glass chamber, and Dual-channel capacitive sensors were placed on their hind paw toes. Once the rats were calm, weight-bearing differences were recorded. To ensure data accuracy, three readings were taken per rat with a 5-min interval, and a total of three rounds were conducted weekly.

### Gait analysis

Automated locomotor pattern analysis was performed utilizing the Cat Walk apparatus (Noldus Information Technology, Wageningen, Netherlands) as described previously [28]. This technology consists of a confined corridor with Light-dispersing glass flooring that illuminates upon paw contact, creating visible footprint patterns. A camera captures 50 to 60 frames per second, and the data is stored for offline analysis using Cat Walk XT 10.6 software. This system enables the evaluation of gait changes and weight-bearing by selecting relevant parameters [29].

### Histological analysis

The H&E staining procedure (Additional File 1: Method S3) can reveal changes occurring at the articular cartilage interface, layering patterns, and chondrocyte characteristics, including quantity and structural features. The safranin-O positive area was measured using the ImageJ analysis platform to quantitatively evaluate the cartilage matrix [30]. At the same time, pathological changes in the synovium were evaluated and scored using hematoxylin and eosin H&E staining.

### TUNEL staining

The TUNEL apoptosis detection kit (Servicebio, Wuhan, China) (Additional file 1: Methods S4) was used to detect the apoptosis cells of chondrocytes in cartilage tissue. Visualization and documentation of apoptotic chondrocytes were facilitated through examination with an upright fluorescence microscopic apparatus (Nikon ECLIPSE, Japan).

### Biochemical parameters in serum

Serum levels of key inflammatory factors—tumor necrosis factor-alpha (TNF-α), IL-1β, and prostaglandin E2 (PGE2)—underwent measurement using commercial immunoassay kits (Enzyme linked Biotechnology Co., Ltd, Shanghai, China) following the recommended procedural guidelines. Oxidative stress biomarker assessment included superoxide dismutase (SOD) and glutathione (GSH) quantification using diagnostic reagents supplied by Elabscience (Wuhan, China), whereas malondialdehyde (MDA) detection employed specialized kits from Nanjing Jiancheng Bioengineering Institute (Nanjing, China). All biochemical procedures were performed according to the suppliers'technical instructions[31].

### Western blot analysis

Protein concentration in the supernatant after lysis was measured using the BCA protein quantification kit (Beyotime, Shanghai, China). Membranes were immunoblotted overnight at 4 °C with primary antibodies against MMP13, COL2A1, MYL3, NDUFA5, NDUFA6, NDUFS6, AMPK, and p-AMPK, followed by HRP-conjugated secondary antibody incubation. Blots were examined with ECL detection reagents and quantified using ImageJ Software. Antibody information is provided in (Additional file 2: Table S2).

### Preparation of DAP and transdermal samples and analysis of chemical profiles

Comprehensive details regarding the preparation of DAP samples and the in vitro transdermal sample preparation are provided in (Additional file 1: Methods S5-6, Additional file 2: Table S3). Ultra Performance Liquid Chromatography-Quadrupole-Time-of-Flight Mass Spectrometry (UPLC-Q-TOF/MS) was utilized to analyze the compounds of the original DAP formula and, in conjunction with Franz diffusion cell method, to identify the transdermal active ingredients. The raw data obtained were processed utilizing Waters MassLynx v4.1 software for analysis, as described in (Additional file 1: Methods S7).

### RNA-sequencing (RNA-seq)

Cartilage from the right knee joint of rats was collected from the most effective DAP-H group, KOA model group, and control group (n = 3) for RNA-seq analysis. RNA-seq was performed by Sinotech Genomics Co., Ltd. (Shanghai, China) using the VAHTS Universal V10 RNA-seq Library Prep Kit for Illumina by Vazyme Biotech Co., Ltd, (Nanjing, China). Sequencing was carried out on the Illumina Nova Seq high-throughput platform. Significant differentially expressed genes (DEGs) were screened with the thresholds of |Fold change|> 1.5 and *P* < 0.05. All raw data have been uploaded to GEO database (Accession No: GSE299712).

### Network construction and analyses

Gene Ontology (GO) functional analysis and Kyoto Encyclopedia of Genes and Genomes (KEGG) pathway enrichment analysis for DEGs were conducted using the DAVID database (https://david.ncifcrf.gov/) [27]. To identify the key genes involved in the regulatory mechanisms of DAP against KOA, a protein–protein interaction (PPI) network was built using the STRING database (https://string-db.org/) with a combined score > 0.7. Node topological features were calculated using CytoHubba, and core targets were screened with the median MCC (6.5) as the cutoff value. Gene co-ex-pression network analysis was established using the “corrplot” and “Hmisc” packages in R. Topological eigenvalues of nodes were calculated using CytoHubba, with Pearson’s correlation coefficient and *P* < 0.05 as the cutoff values.

### Quantitative real-time PCR (qPCR)

Total RNA was extracted using TRNzol reagent (TIANGEN, Beijing, China) and reverse-transcribed using a cDNA synthesis kit (TIANGEN, Beijing, China). The expression of targets was determined in triplicates using SYBR (Takara, Japan) on a Roche LightCycler 480 system. Relative gene expression was calculated using the 2^− ΔΔCT^ method and normalized to β-actin. Primer sequences are detailed in (Additional file 2: Table S4).

### Immunohistochemical staining (IHC)

For assessment of DAP effects on candidate target localization and expression [32]. (Additional file 1: Methods S8), we applied specific rabbit antibodies against NDUFA5, NDUFA6, NDUFS6, p-AMPK, AMPK, MYL3, Aquaporin 1 (AQP1), Aquaporin 3 (AQP3), and Aquaporin 4 (AQP4). Quantification of staining intensity involved measuring average optical density (AOD) using ImageJ software across three separate fields of view per sample (n = 3). Details regarding the antibodies are provided in( Additional file 2: Table S2).

### Isolation and cultivation of primary chondrocytes

Primary chondrocytes were isolated from 4-week-old male SD rats (120 ± 10 g) [33]. After euthanasia, cartilage tissues were aseptically collected, minced, and washed with cold PBS. Sequential enzymatic digestion was performed using 0.25% trypsin (40 min) followed by 0.2% type II collagenase (37 °C, 6 h). Cells were filtered through 70 μm strainers, centrifuged at 1000 r/min, and cultured in DMEM containing 10% FBS and 1% penicillin–streptomycin.

### Cell viability assays

A suspension of chondrocytes was prepared, then seeded in 96-well plates (1.0 × 10^4^ cells/well) and allowed to attach during overnight incubation. The Cell Counting Kit-8 enabled assessment of cellular viability as detailed (Beyotime, Shanghai, China) (Additional file 1: Methods S9). The details of the 19 primary transdermal compound standards in DAP are presented in (Additional file 2: Table S5)**.**

### Cell treatment

Chondrocytes were treated with 19 transdermal ingredients at a concentration of 50 μM. The treatment protocol was as follows [34]: chondrocytes were divided into 21 groups, with 19 transdermal ingredients co-cultured with 10 ng/mL IL-1β. As reported, IL-1β functions as an established inducer of osteoarthritic phenotype in chondrocytes, frequently employed for simulating pathological conditions in laboratory settings [35]. For a Duration of 24 h, all experimental groups excluding controls underwent cultivation in serum-free medium supplemented with 10 ng/mL IL-1β.

### Co-immunoprecipitation (Co-IP)

Co-IP was performed to explore the interactions between MYL3 and AMPK. Cells were lysed using IP lysis buffer (Beyotime, Shanghai, China), and the supernatant was collected after centrifugation at 12,000 r/min. The cell lysates were incubated overnight at 4 °C with antibodies targeting MYL3 (Proteintech, Wuhan, China) and normal rabbit IgG (Beyotime, Shanghai, China). The lysates were then combined with protein A/G agarose beads (Cell Signaling Technology, USA) and incubated for 2 h. The resulting precipitates were washed with ice-cold IP buffer and analyzed by Western blot.

### Measurement of ROS levels

Intracellular ROS quantification employed fluorescence probe methodology utilizing a DCFH-DA-based detection kit (Beyotime, Shanghai, China). An Olympus microscope (Tokyo, Japan) facilitated visual data acquisition, while Image software enabled comprehensive analysis of three randomly captured fields [36].

### MMP assay

Chondrocytes were stained with JC-1 (Beyotime, Shanghai, China) to assess MMP. Following drug treatment, the JC-1 staining working solution was added and mixed thoroughly. The cells were incubated at 37 °C for 20 min. Observations were conducted using an Olympus microscope (Tokyo, Japan), and detailed analysis was performed on 3 randomly selected images using Image software.

### Measurement of mitochondrial ATP content

ATP levels in chondrocytes were measured using a commercial ATP detection kit (Beyotime, China). Briefly, 100 μL working solution was added to each well, followed by 5-min incubation at room temperature. Then, 20 μL sample or standard was added, mixed thoroughly, and RLU was measured after 2 s using a chemiluminescence analyzer.

### Transmission electron microscopy (TEM)

Chondrocytes were fixed in 2.5% glutaraldehyde at 4 °C for 12 h, followed by a 1-h treatment with 2% osmium tetroxide. The cells were then stained with 2% uranyl acetate and dehydrated by sequential immersion in ethanol, from 30 to 100%. Specimens underwent acetone (100%) washing with 20-min agitation before epoxy resin infiltration, polymerization, ultrathin sectioning, and ultrastructural examination using Hitachi™ TEM (Tokyo, Japan).

### Molecular docking

AutoDockTools 1.5.7 software enabled computational molecular docking analyses to elucidate interaction patterns between DAP active ingredients and key protein targets. Component three-dimensional structural data originated from PubChem repository, whereas target protein crystallographic coordinates were acquired from RCSB Protein Data Bank (http://www.rcsb.org/). The target structure was edited using PyMOL 2.3.0, and the ingredients were treated as ligands for semi-flexible docking. Interactions and binding modes were evaluated, and were visualized using PyMOL.

### Cellular thermal shift assay (CETSA)

After 24 h of treatment with DMSO and 1.5% dicaffeoylquinic acid in chondrocytes, heat the samples in a PCR machine at the specified temperatures (40, 43, 46, 49, 52, 56, 60, 64, 68, and 72 °C) for 5 min, with subsequent rapid cooling at 4 °C lasting 3 min. Next, use ultrasound treatment to lyse the cells. Centrifuge the cell suspension at 4 °C at 14,000 r/min for 30 min. After centrifugation, add 5 × loading buffer to the supernatant, mix thoroughly, and Heat at 100 °C for 8 min for protein blotting detection.

### Surface plasmon resonance (SPR) assay

Biomolecular interaction analysis employed a Biacore 8 K system (Cytiva, Sweden) following methodology previously established by our group with full protocol details available [37] (Additional file 1: Methods S10). Kinetic parameters including association and dissociation rate constants were determined through global data fitting to a 1:1 Langmuir interaction model utilizing Biacore Insight evaluation software suite (Cytiva, Marlborough, MA, USA).

### Flow cytometry

Digest the cells to be detected with trypsin, collect the cells in a centrifuge tube, wash the collected cells with PBS and centrifuge to remove the supernatant. Resuspend cells with 195 μ l of Annexin V-FITC binding solution, add 5 μL of Annexin V-FITC and 10 μL of propidium iodide (PI) staining solution, mix well, incubate at room temperature in the dark for 10-min, and detect using a flow cytometer.

### Detection of mitochondrial complex 1 content

Cellular material underwent harvesting, washed with PBS, centrifuged, a with subsequent collection of supernatant fraction for analytical processing. Reference standards and experimental samples were dispensed into designated microplate wells followed by a 30-min incubation at physiological temperature (37 °C). Measure the absorbance at 450 nm according to manufacturer-specified protocol parameters.

### Statistical analyses

Data are presented as mean ± standard deviation (SD). Data analysis was conducted using GraphPad Prism (v8.4.3). Parametric or non-parametric statistical analyses were employed based on the normal distribution testing results. All experimental data underwent analysis using a one-way (ANOVA) with a post-hoc Tukey test. *p* < 0.05 was considered statistically significant.

## Results

### DAP treatment alleviates disease severity in the KOA rat model

**Fig. 1:**
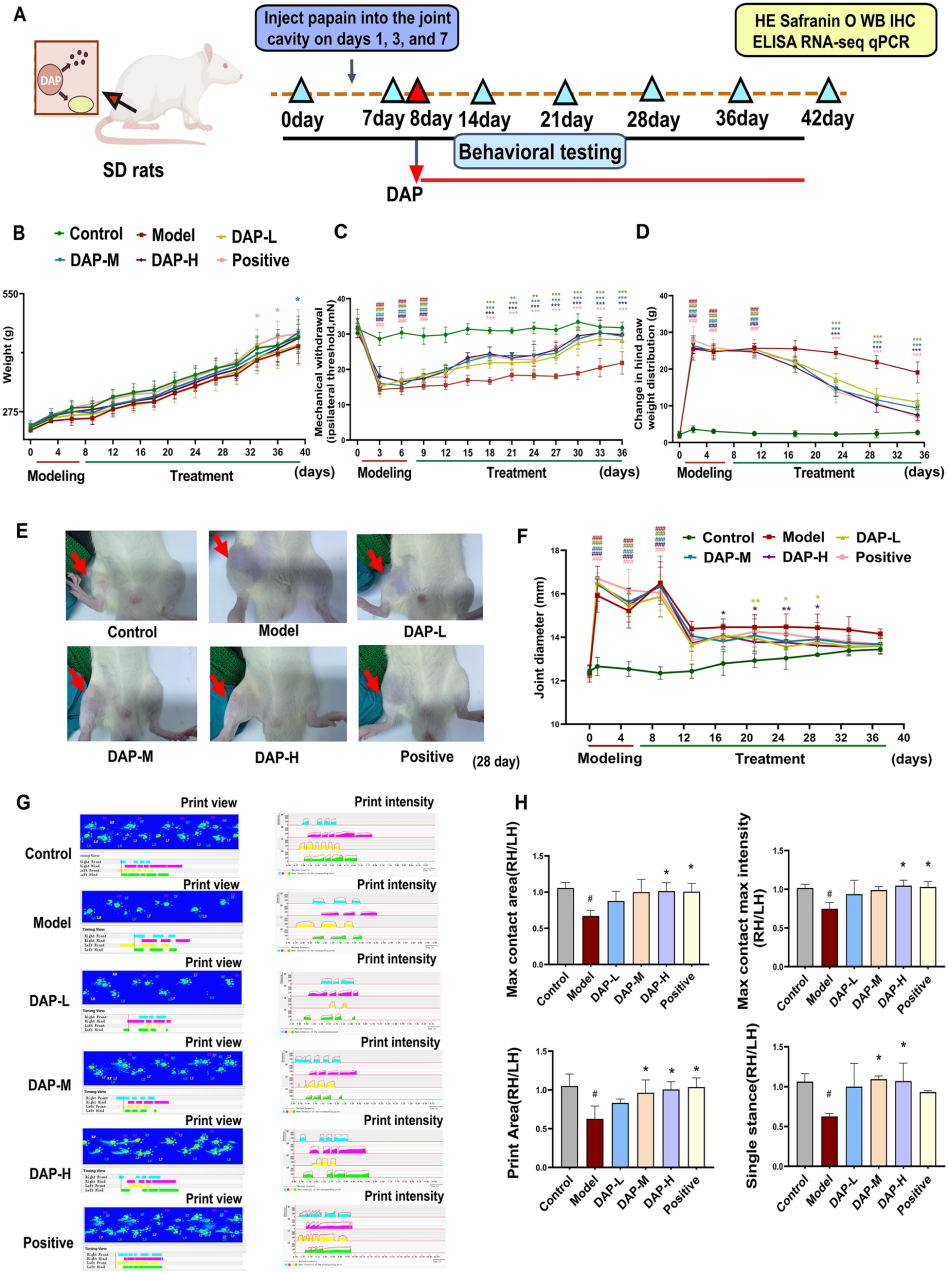
DAP treatment alleviates arthritis severity in a KOA rat model. **A** Experimental paradigm timeline schematic. **B** Body weight changes in each group (n = 6–12). **C** Mechanical-induced hyperalgesia pain thresholds across groups (n = 6). **D** Weight distribution between left and right hindlimbs in various groups (n = 6). **E**, **F** Representative images of affected joints and changes in knee joint diameter across groups (n = 6). **G** Representative Cat Walk gait analysis showing Timing view, Print view, and Print intensity for each group. **H** Statistical charts of Max contact area, Max contact intensity, Print Area, and Single stance, with data normalized using the LH/RH formula to control for confounding factors (n = 3). ^#^* P* < 0.05, ^##^* P* < 0.01, ^###^* P* < 0.001, comparison with the control group, * P < 0.05, ** P < 0.01, *** P < 0.001, comparison with the model group

After the establishment of KOA induced by papain, rats underwent DAP treatment. Behavioral assessments were performed to evaluate the therapeutic effect of DAP, as shown in (Fig. 1A). The body weight of all rats increased with age throughout the experimental period. The highest weight was maintained in the control group, while the lowest was observed in the KOA group, indicating that DAP improved weight loss in KOA rats (Fig. 1B). To assess pain relief, mechanical withdrawal threshold was measured. From day 11 post-treatment until the end of the study, the mechanical withdrawal threshold increased in the DAP-L, DAP-M, and DAP-H groups, suggesting pain relief (Model *vs*. Control, all *P* < 0.001, DAP *vs*. Model, *P* < 0.001, Fig. 1C). Weight-bearing asymmetry between hindlimbs diminished in DAP-L, DAP-M, and DAP-H groups starting from post-treatment day 16 (Model *vs*. Control, all *P* < 0.001, DAP *vs*. Model, *P* < 0.001, Fig. 1D). To evaluate the effect of DAP on joint edema, knee joint swelling was measured. The DAP groups showed reduced joint swelling from day 11 post-treatment (Model *vs*. Control, all *P* < 0.001, DAP *vs*. Model, *P* < 0.05, Fig. 1E, F). Gait analysis was conducted to assess movement disorders and pain. The model group exhibited significantly lower maximum contact area, maximum contact intensity, print area, and single stance duration, which were all improved by DAP-L, DAP-M, and DAP-H treatments (Model *vs*. Control, all *P* < 0.05, DAP *vs*. model, *P* < 0.05, Fig. 1G, H). These findings suggest that DAP treatment significantly alleviates gait abnormalities and pain in KOA rats, consistent with the results of mechanical pain testing.

### DAP effectively promotes cartilage matrix synthesis and reduces degradation

To evaluate the in vivo cartilage-protective effects of DAP, a comprehensive analysis of cartilage tissues was performed. Apoptotic activity was assessed using TUNEL staining, while tissue morphology was examined with H&E staining. Safranin O-fast green methodology enabled visualization of proteoglycan distribution and ECM structural integrity. Western blot analysis quantified key molecular markers involved in cartilage synthesis and degradation.

**Fig. 2:**
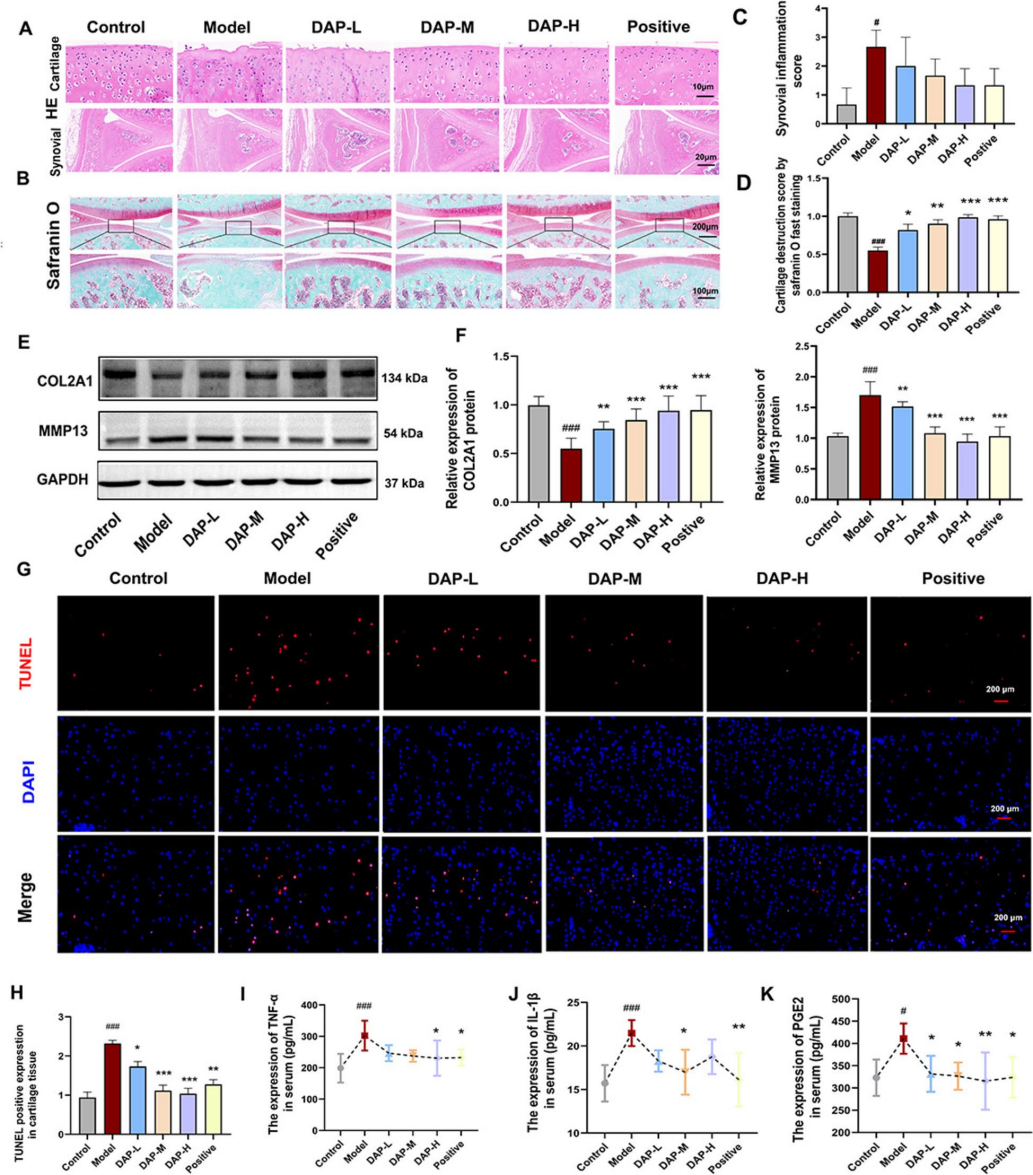
DAP promotes cartilage matrix synthesis and reduces degradation. **A** H&E staining of knee joints and synovial from different groups. **B** Safranin O fast staining of knee joint cartilage tissues in various groups. **C** Quantitative analysis of the synovial inflammation scores in different groups (n = 3). **D** Cartilage destruction scores assessed by Safranin O fast staining in different groups (n = 3). **E**, **F** Expression levels of COL2A1 and MMP13 proteins in knee joint cartilage tissues across groups (n = 3). **G** Representative TUNEL staining images of knee joint cartilage tissues in different groups. **H** TUNEL positive expression levels in cartilage tissues from various groups (n = 3). **I**–**K** Serum levels of TNF-α, IL-1β, and PEG2 across groups (n = 6). Data are expressed as mean ± SD. ^#^*P* < 0.05, ^##^*P* < 0.01, ^###^*P* < 0.001, comparison with the control group, * P < 0.05, ** P < 0.01, *** P < 0.001, comparison with the model group

Histopathological evaluation of synovitis demonstrated that synovial cells in the normal group were regularly and uniformly arranged, with no detectable infiltration of inflammatory cells. In contrast, the model group exhibited marked infiltration of inflammatory cells. Following treatment with anti-swelling and analgesic patches, the treated group showed reduced inflammatory cell infiltration, accompanied by relatively loose cellular arrangement (Fig. 2A,C). Histopathological assessment through H&E and safranin O-fast green techniques demonstrated cartilage thinning, structural compromise, and chondrocyte disorganization in KOA model group relative to control group. The cartilage indicated lighter orange-red staining, indicating a thinner matrix and significant damage, along with inflammatory cell infiltration and sparse subchondral bone trabeculae. Treatment with DAP induced varying degrees of recovery, where higher dosages significantly mitigated cartilage damage (Model *vs*. Control, all *P* < 0.001; DAP *vs*. Model, *P* < 0.05, Fig. 2A,B–D). This treatment also reversed pathological changes, which correlated with changes in cartilage matrix markers (COL2A1 and MMP13) (Model vs. Control, all *P* < 0.001; DAP *vs*. Model, all *P* < 0.01, Fig. 2E, F). Apoptotic chondrocyte populations, visualized via TUNEL methodology, exhibited significant elevation in KOA model groups compared with controls, progressively declining with increasing DAP dosage (Model *vs*. Control, *P* < 0.001; DAP *vs*. Model, *P* < 0.05, Fig. 2G, H). Furthermore, DAP significantly decreased serum levels of inflammatory cytokines (Fig. 2I–K). The observed findings indicate DAP confers chondroprotection against degradative and inflammatory processes in KOA pathology.

### Gene expression profile-based analysis reveals that DAP may alleviate KOA by regulating material and energy metabolism, and the immune-inflammatory system

**Fig. 3:**
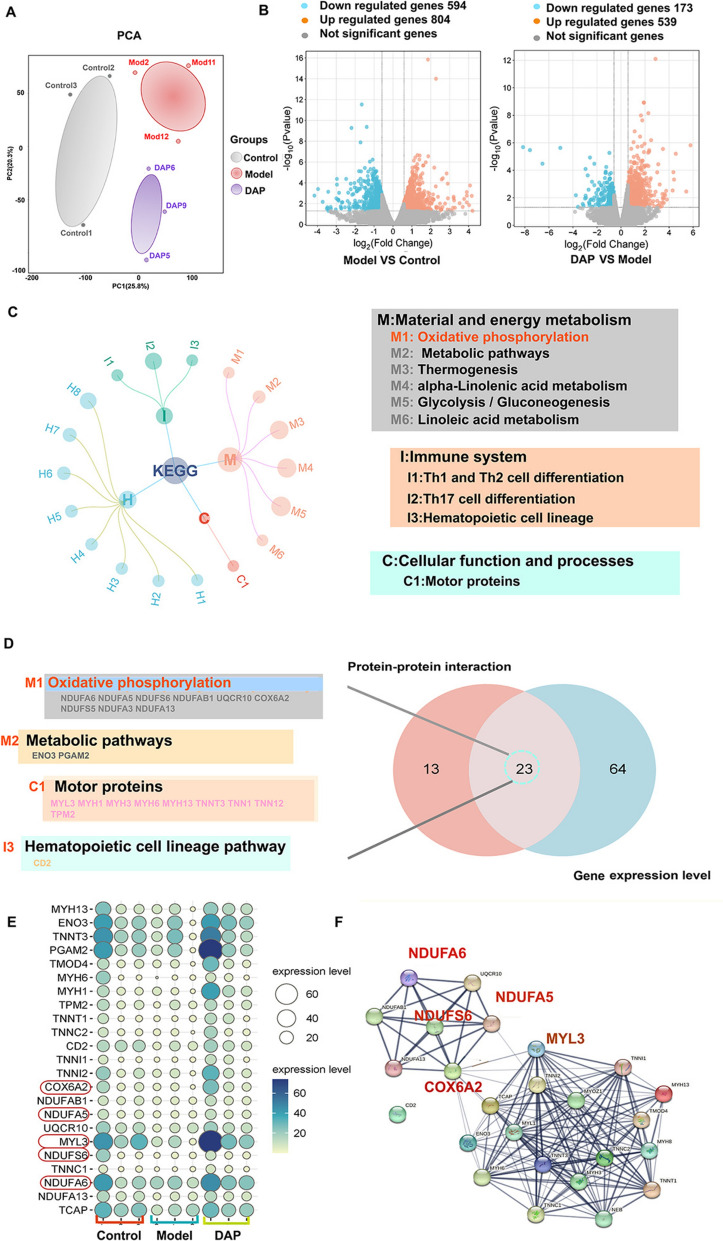
Bioinformatics analysis of KOA rats’ cartilage transcriptome (n = 3). **A** PCA analysis of control, model, and DAP-H groups. **B** Volcano plot analysis illustrating differential gene expression in control, model, and DAP-H groups, with significant genes marked (|Fold Change |> 1.5, *P* < 0.05, blue and red indicate down- and up-regulated expression, respectively). **C** KEGG pathway enrichment analysis illustrating the effects of DAP on KOA. **D** Identification of intersection target sets from PPI and gene co-ex-pression network analysis, identifying a total of 23 genes. **E** Balloon plot visualization showing expression levels of 23 genes (Circle size and color depth represent gene expression levels). **F** Protein interaction analysis of 23 proteins using the STRING database

RNA-seq was conducted on rat articular cartilage specimens from control, model, and DAP intervention cohorts to elucidate underlying molecular pathways affected by treatment. Principal component analysis (PCA) showed clear differentiation between the control, model, and DAP groups (Fig. 3A). A total of 1,398 DEGs were identified between the model and control groups, and 712 DEGs were identified between the DAP and model groups, using criteria of |fold change|> 1.5 and *P* < 0.05 (Fig. 3B). Among these, 206 DEGs were reversed by DAP treatment (Additional file 3: Fig. S1A).

By performing GO enrichment analysis of 206 DEGs revealed that they were localized in mitochondria and closely related to mitochondrial respiratory chain complex I, mitochondrial inner membrane and mitochondrial membrane. The biological processes were associated with mitochondrial electron transport, NADH to ubiquinone and mitochondrial ATP synthesis coupled proton transport. The molecular functions were closely related to NADH dehydrogenase (ubiquinone) activity and sodium channel regulator activity (Additional file 3: Fig. S1B-D). KEGG enrichment analysis revealed that the DEGs were mainly enriched in material and energy metabolism, cellular function and processes, and imbalance of"immune-inflammation"system regulation. Inflammation is a key driver in the development of KOA, with both the innate and adaptive immune systems playing significant roles. The increase in inflammatory cell levels can speed up the degradation of cartilage. Moreover, antigens released from damaged joints can further incite inflammatory reactions by activating inflammasomes [38]. It is worth noting that the oxidative phosphorylation pathway showed the most significant enrichment among these DEGs (Fig. 3C).

Topological node characteristics were computed via CytoHubba to elucidate crucial genetic elements within DAP's regulatory activity against KOA, yielding 36 significant targets (with a median MCC value of 6.5). A gene co-ex-pression network analysis was established using the"corrplot"and"Hmisc"packages in R, yielding 87 key targets (with a median degree value of 172). Ultimately, 23 key genes were identified (Fig. 3D–F). KEGG pathway analysis showed that these genes were mainly enriched in the oxidative phosphorylation signaling pathway, including NDUFA6, NDUFA5, NDUFS6, and COX6A2. Among them, NDUFA6, NDUFA5, and NDUFS6 are critical for mitochondrial complex I assembly and function [39]. As a major gene of mitochondrial complex IV, COX6A2 codes for an isoform of a cytochrome c oxidase subunit. The expression of COX6A2 enhances oxidative stress and impairs the maturation of its morphological and functional characteristics [40]. Additionally, MYL3, which is closely related to cartilage, was also identified [41].

### DAP may alleviate cartilage degeneration by rescuing mitochondrial complex 1/AMPK/MYL3 signaling pathway

**Fig. 4:**
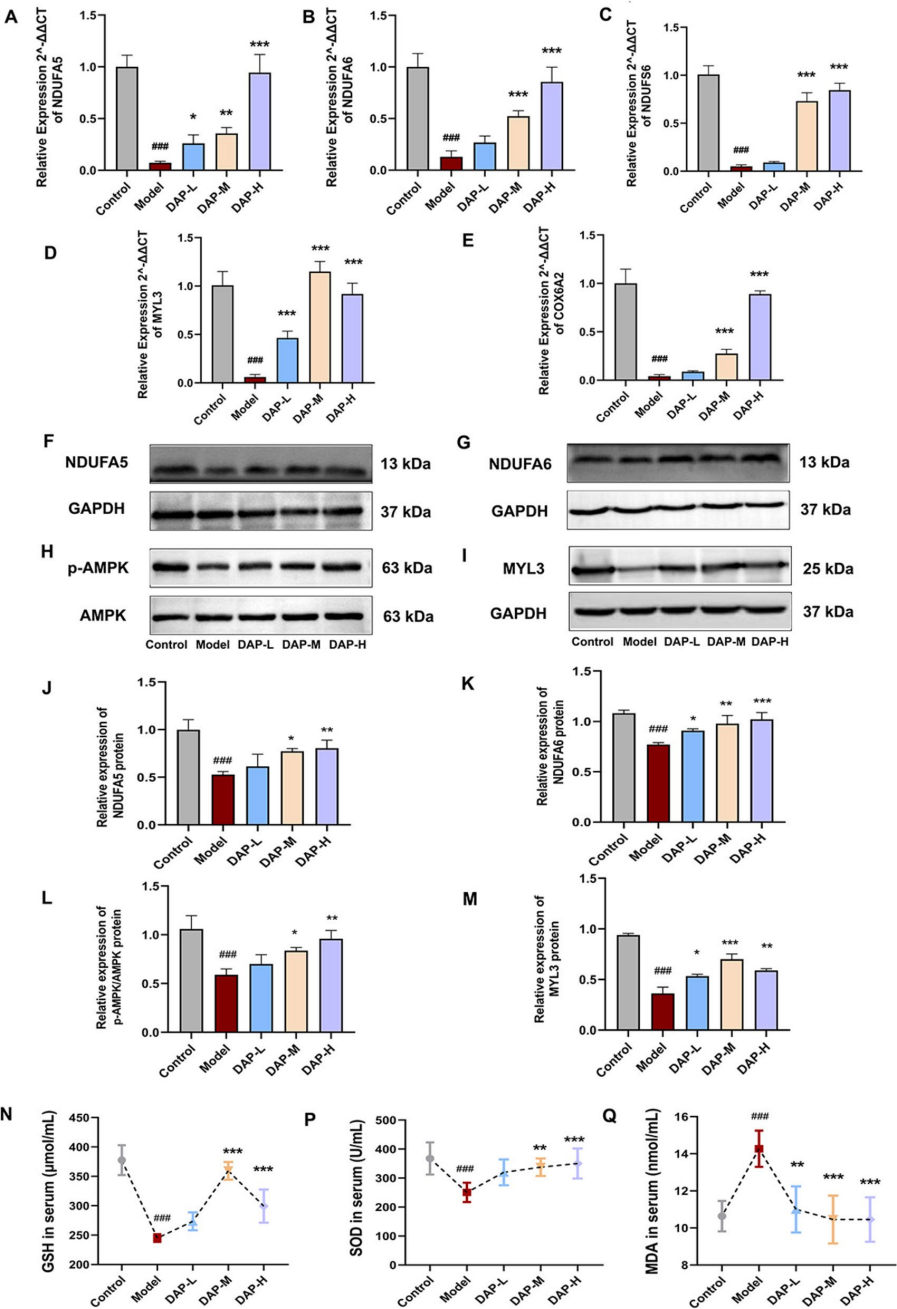
In vivo experiments indicate mitochondrial complex I, AMPK, and MYL3 as effective targets of DAP in alleviating KOA severity. **A**–**E** qPCR analysis of gene expression for NDUFA5, NDUFA6, MYL3, NDUFS6 and COX6A2 (n = 6). **F**–**M** Western blot analysis showing protein expression of NDUFA5, NDUFA6, AMPK, p-AMPK, and MYL3, and their statistical results (n = 3). **N**–**P** Serum levels of GSH, SOD, and MDA (n = 6). Data are expressed as mean ± SD. ^**#**^*P* < 0.05, ^**##**^*P* < 0.01, ^**###**^*P* < 0.001, comparison with the control group, *P < 0.05, **P < 0.01, ***P < 0.001, comparison with the model group

**Fig. 5:**
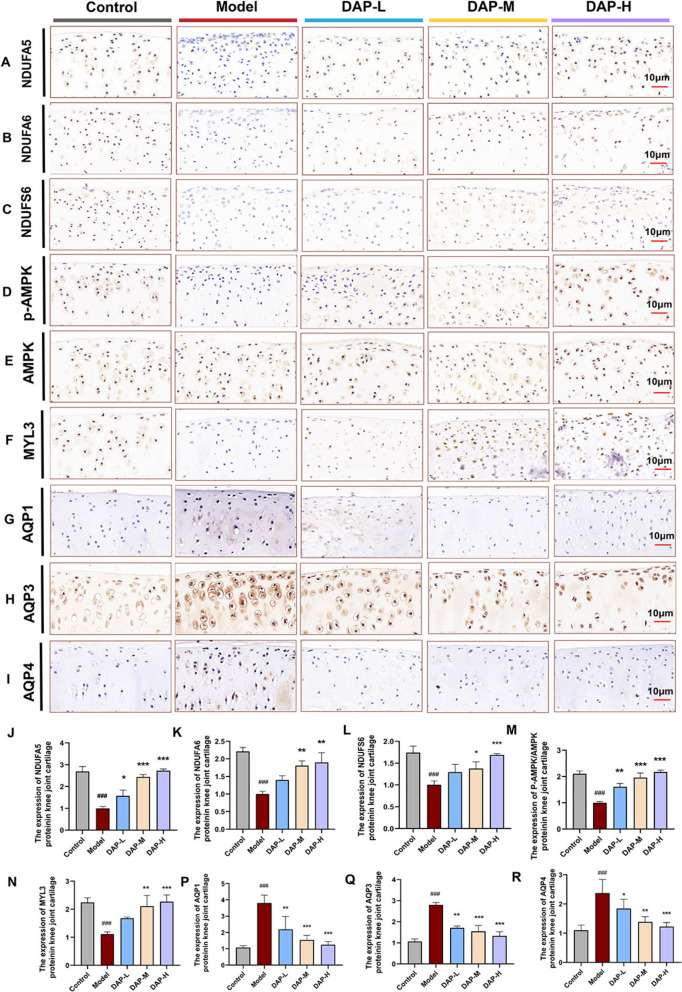
DAP effectively reduces edema and cartilage degeneration through antioxidant stress response. **A**–**Q** IHC analysis of protein expression for NDUFA5, NDUFA6, NDUFS6, p-AMPK, AMPK, MYL3, AQP1, AQP3, AQP4, and their statistical results. Data are expressed as mean ± SD (n = 3). ^#^*P* < 0.05, ^##^*P* < 0.01, ^###^*P* < 0.001, comparison with the control group, *P < 0.05, **P < 0.01, ***P < 0.001, comparison with the model group

To validate the RNA-seq results, five DEGs were confirmed by qPCR analysis in cartilage tissues from KOA rats in the control, model, and DAP treatment groups. The mRNA levels of mitochondrial complexes (NDUFA6, NDUFA5, NDUFS6, COX6A2) and MYL3 were downregulated in KOA but restored by DAP, consistent with the RNA-seq findings (Fig. 4A–E). Protein levels of NDUFA5, NDUFA6, NDUFS6 and MYL3 were significantly lower in KOA rats compared to controls (Model *vs*. Control, *P* < 0.001; DAP *vs*. Model, *P* < 0.05, Figs. 4F, G, I–K, M, 5A–C, F, J–L, N). DAP treatment effectively reversed these abnormalities.

Elevated ROS can promote cartilage degeneration by damaging collagen and proteoglycans and activating ECM metalloproteinases. However, DAP enhances complex I synthesis, reducing ROS production. To assess whether DAP inhibits KOA progression by suppressing ROS, we measured serum levels of MDA, SOD, and GSH. DAP treatment significantly decreased MDA levels in a dose-dependent manner and increased SOD and GSH activities in KOA rats (Fig. 4N–P).

A significant decrease in p-AMPK/AMPK protein expression levels was detected in KOA cartilage tissue, DAP treatment effectively increased the expression level of p-AMPK/AMPK protein (Model vs. Control, *P* < 0.001; DAP vs. Model, *P* < 0.05, Figs. 4H, L, 5D, E, M). Administration of DAP successfully restored these alterations to normal levels. To elucidate the interaction between AMPK and MYL3, protein-to-protein docking was used to predict their potential interaction (Additional file 3: Fig. S2A). Co-IP experiments further confirmed that, compared with the control group, the expression of AMPK and MYL3 proteins was significantly downregulated in the model group. Following drug administration, the expression of these proteins was reversed, as demonstrated in (Additional File 3: Figure S2B–D). Moreover, treatment with DAP significantly reduced the protein levels of AQP1, AQP3, and AQP4 (Model *vs*. Control, *P* < 0.001; DAP *vs*. Model, *P* < 0.05, Fig. 5G–I, O–Q).

### Chemical ingredients and skin-penetrating constituents of DAP identified via UPLC-Q-TOF/MS system

**Fig. 6:**
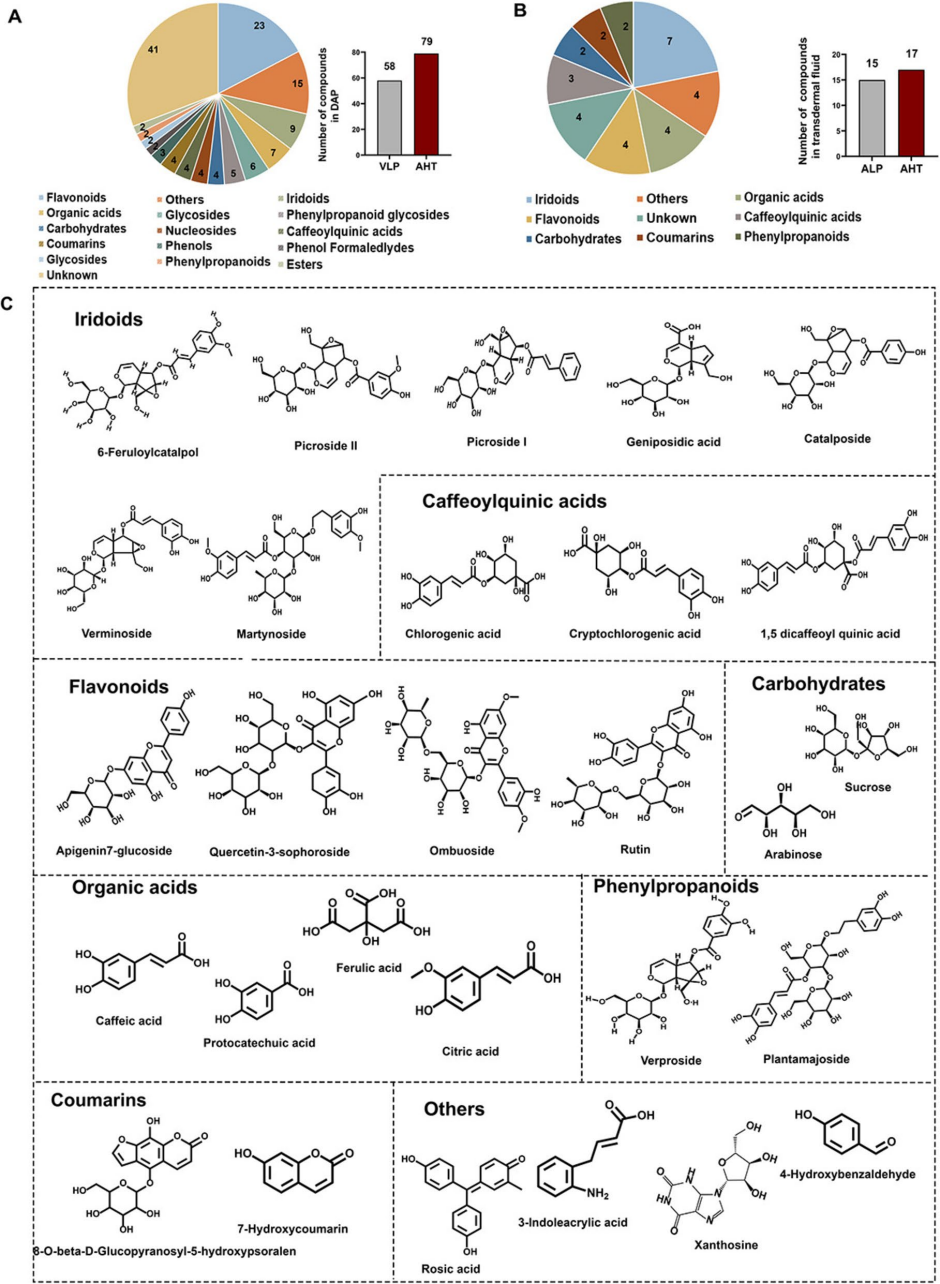
Identification of chemical ingredients and skin-penetrating constituents of DAP using the UPLC-Q-TOF/MS system. **A** Structural classification and count of chemical ingredients in DAP. **B** Structural classification and count of transdermal compounds in DAP. **C** Structures of transdermal compounds in DAP

UPLC-Q-TOF/MS methodology generated high-resolution mass spectral profiles for DAP, yielding BPI chromatographic patterns illustrated in( Additional file 3: Fig. S3 ). The MS data were processed with the UNIFI filtering platform and matched against standard fragment information. Further verification was conducted using the ETCM2.0 database (http://www.tcmip.cn/ETCM2/front/#/), resulting in the identification of 51 previously unrecognized compounds. A total of 92 ingredients were identified. Detailed MS information for these compounds is summarized in (Additional file 2: Table S6). The chemical structures were confirmed and classified based on accurate mass spectrometry data and relevant literature (Fig. 6A). Notably, 28 compounds from the DAP prescription were found to be capable of skin penetration, as shown in (Additional file 3: Fig. S4, Fig. S5, and Additional file 2: Table S7). Key transdermal ingredients identified include picroside II, picroside I, verproside, geniposidic acid, 6-feruloylcatalpol, and catalposide from VLP, along with chlorogenic acid, caffeic acid, and 1,5-dicaffeoylquinic acid from AHT seeds. The classification and categorization of these compounds are illustrated in Fig. 6B, with structural formulas presented in Fig. 6C.

### Screening transdermal active ingredients with anti-inflammatory and cartilage protective effects

**Fig. 7:**
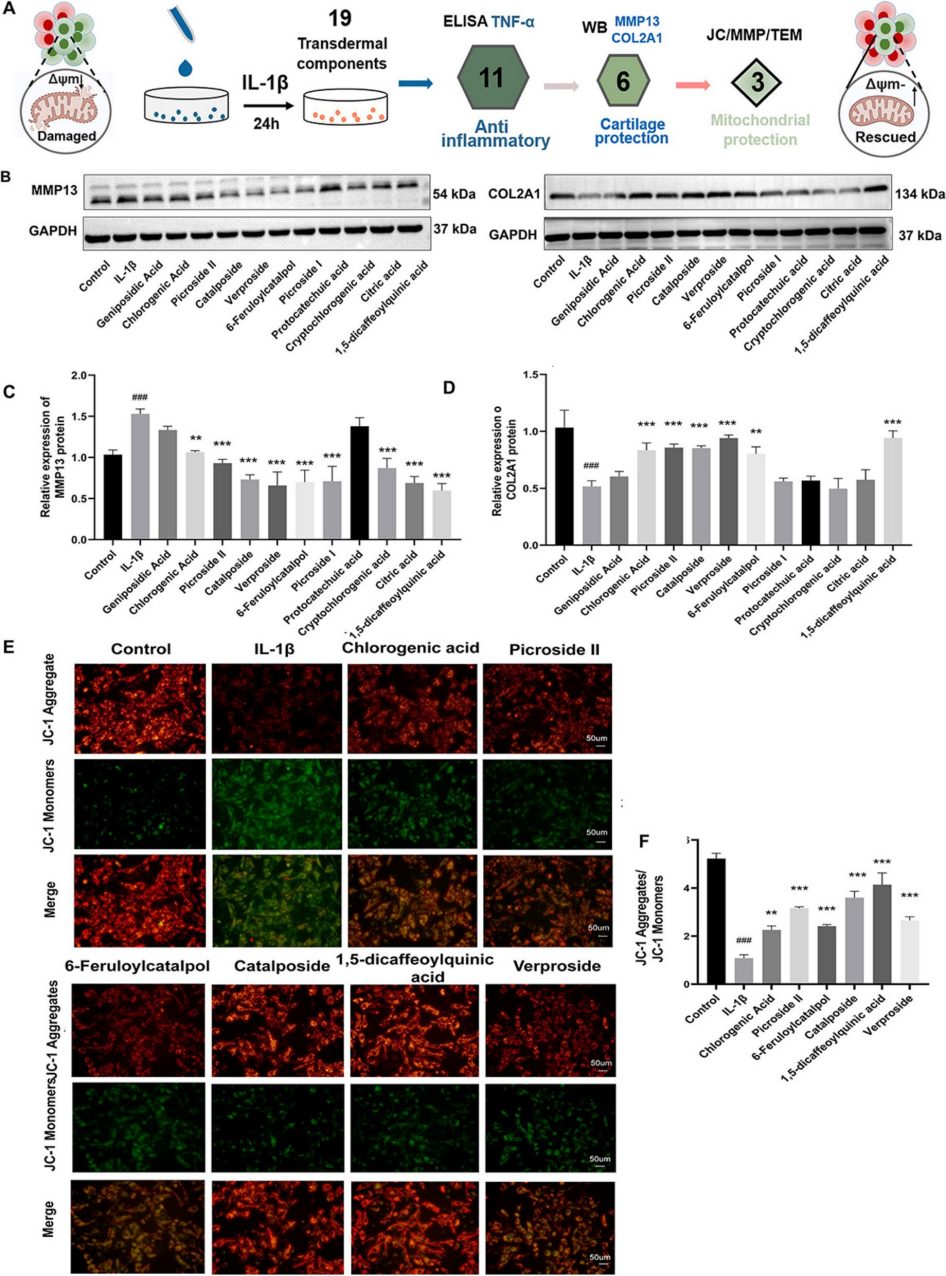
Screening active ingredients for anti-inflammatory and cartilage protective effects. **A** Schematic timeline of the cellular experimental paradigm. **B**–**D** Expression levels of MMP13 and COL2A1 proteins following treatment with 11 active ingredients (n = 3). **E** Changes in MMP detected by JC-1 probe staining. **F** Membrane potential analysis based on the ratio of red to green fluorescence (n = 3). Data are expressed as mean ± SD. ^#^*P* < 0.05, ^##^*P* < 0.01, ^###^*P* < 0.001, comparison with the control group, *P < 0.05, **P < 0.01, ***P < 0.001, comparison with the IL-1β group

This study assessed the viability of chondrocytes exposed to 19 transdermal ingredients at varying concentrations (0, 25, 50,100 μM) for 24 h (Additional file 3: Fig. S6). Only 19 transdermal ingredients were purchased, and it was determined that they were non-toxic at concentrations of 25–50 μM, leading to a final dose of 50 μM. TNF-α levels were measured using ELISA, and 11 ingredients with anti-inflammatory activity were identified, ranking in the top 60% (Additional file 3: Fig. S7). Protein expression profiling via Western blot demonstrated that IL-1β treatment reduced COL2A1 levels while elevating MMP13 expression relative to untreated controls. In contrast, treatment with 1,5-dicaffeoylquinic acid, 6-feruloylcatalpol, picroside II, verproside, catalposide, and chlorogenic acid significantly restored the expression levels of COL2A1 and MMP13 (Fig. 7A–D). Mitochondrial function assessment revealed that IL-1β group significantly decreased JC-1 polymer (red) fluorescence while increasing monomer (green) fluorescence (*P* < 0.001, Fig. 7E, F). All 6 transdermal compound interventions reversed these changes, restoring JC-1 polymer fluorescence and reducing monomer fluorescence ( *P* < 0.001, Fig. 7E, F).

### 1,5-dicaffeoylquinic acid, verproside, catalposide may be the representative bioactive ingredients in DAP

**Fig. 8:**
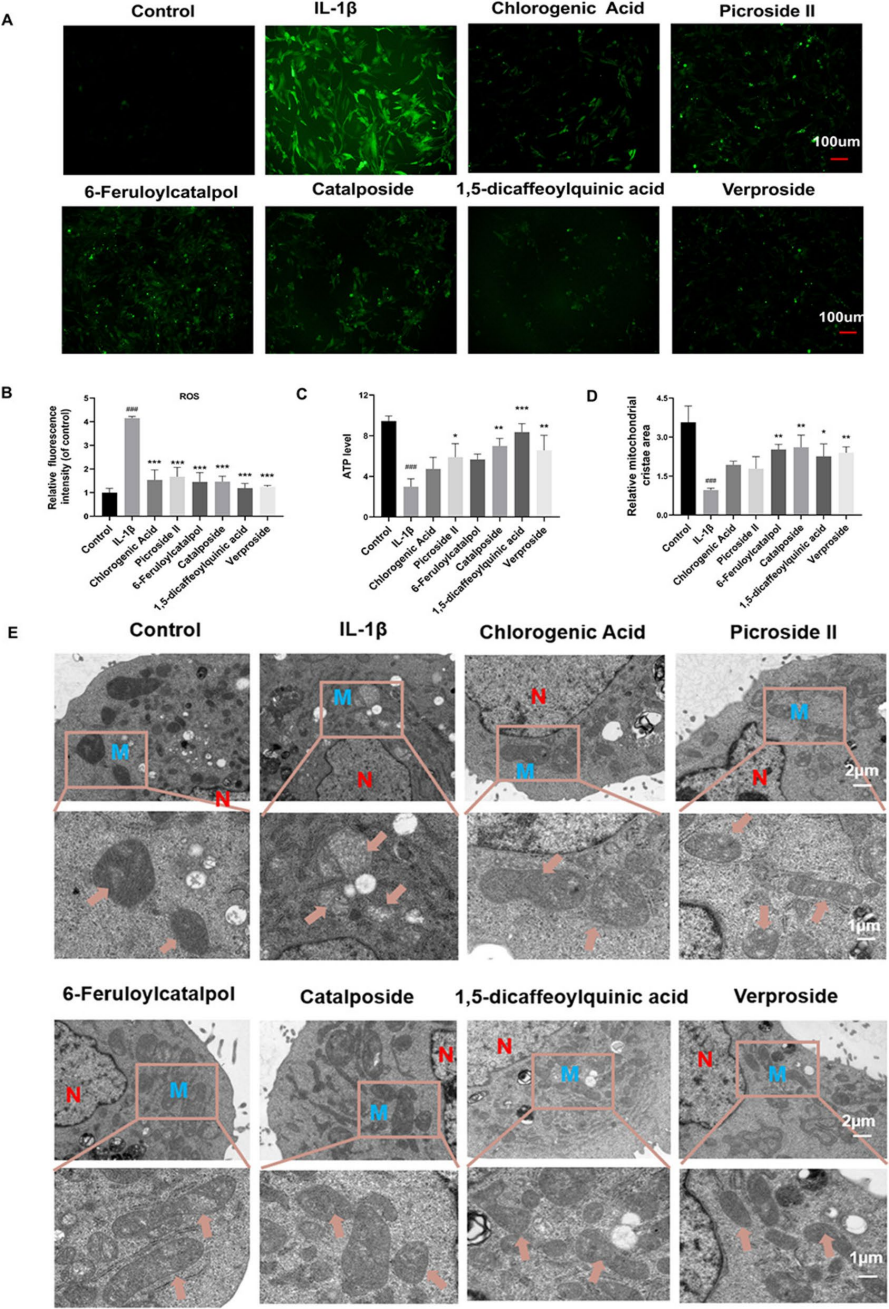
Impact of DAP active ingredients on mitochondrial function induced by IL-1β. **A**–**B** ROS content in chondrocytes was measured using the DCFH-DA fluorescent probe for each group (n = 3). **C** Impact of active ingredients on ATP content (n = 3). **D**, **E** TEM was used to observe the structural damage to chondrocyte mitochondria induced by IL-1β (M: Mitochondria, N: Nucleus, n = 3). Statistical chart of relative Mitochondrial cristae area in each group. Data are expressed as mean ± SD, ^#^*P* < 0.05, ^##^*P* < 0.01, ^###^*P* < 0.001, comparison with the control group, *P < 0.05, **P < 0.01, ***P < 0.001, comparison with the IL-1β group

To further evaluate the effect of transdermal active ingredients in DAP on chondrocyte mitochondria in vitro, we investigated the effects of 6 active ingredients on ROS levels, ATP content, MMP and mitochondrial morphology in an IL-1β-induced chondrocyte mitochondrial dysfunction model. Changes in MMP directly affect intracellular ROS levels. Figure 8A, B illustrates that IL-1β group significantly elevated intracellular DCFH-DA fluorescence intensity in chondrocytes (*P* < 0.001). In contrast, the relative fluorescence intensity of DCFH-DA in cells treated with the 6 transdermal active ingredients decreased significantly (*P* < 0.001), indicating that these active ingredients reduced the ROS increase induced by IL-1β. As shown in Fig. 8C, ATP content in chondrocytes induced by IL-1β decreased significantly compared to the control group (*P* < 0.001). In comparison to the IL-1β group, treatment with 6 active ingredients resulted in varying degrees of increase in ATP content. Notably, 4 transdermal ingredients demonstrated statistically meaningful enhancement of ATP levels: 1,5-dicaffeoylquinic acid, verproside, catalposide and picroside II (*P* < 0.001, *P* < 0.01, *P* < 0.01, *P* < 0.05). Transmission electron microscopy further confirmed these findings. As shown in Fig. 8D, E, mitochondria in the control group were abundant, short rod-shaped, with intact mitochondrial ridges and a uniform matrix. In the IL-1 β group, severe mitochondrial damage was observed, characterized by sparse matrix, weak dissolution, swelling, spinal collapse, and blurred membrane structure, with significantly reduced mitochondrial crest area. In contrast, treatment groups exhibited varying degrees of mitochondrial recovery, with reduced damage, a more uniform matrix, parallel ridge arrangement, decreased swelling, and intact membranes. Notably, verproside, catalposide, 6-feruloycatalpol, and 1,5-dicaffeoylquinic acid significantly increased the mitochondrial cristae area, indicating an improvement in mitochondrial morphology. These results suggest that verproside, catalposide, and 1,5-dicaffeoylquinic acid may act as protective active ingredients against oxidative stress.

**Fig. 9:**
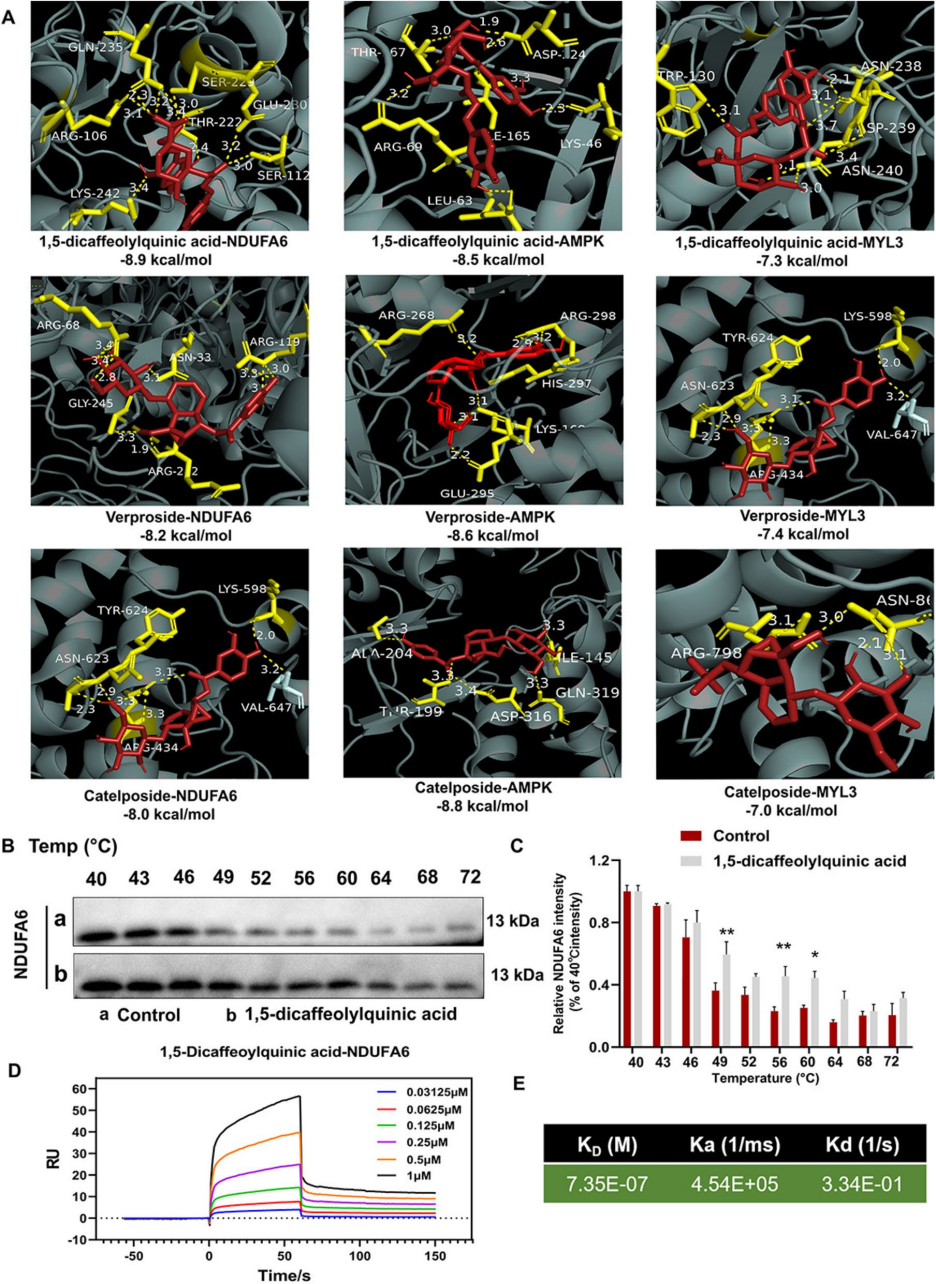
Docking of 1,5-dicaffeoylquinic acid, verproside, and catalposide with key target proteins. **A** Three-dimensional interaction maps and docking binding energies of 1,5-dicaffeoylquinic acid, verproside, and catalposide with NDUFA6 (PDBID: 8bed), AMPK (PDBID: 6c9f)), and MYL3 (PDBID: 8act) protein complexes, respectively. **B** CETSA of NDUFA6 protein that were pretreated with DMSO or 1,5-dicaffeoylquinic acid (50 μM) and treated at multiple temperature, analyzed by western blot. **C** The quantification of NDUFA6 expression was determined as the relative band intensity of that at 40 °C incubation (n = 3). **D**, **E** The interaction between1,5-dicaffeoylquinic acid and NDUFA6 was quantified using an SPR assay, detailing the dissociation constant, binding rate constant, and dissociation rate constant. Data are expressed as mean ± SD, * *P* < 0.05, ** *P* < 0.01, compared with the control group

To confirm the binding interactions between the three transdermal active ingredients and their key targets—mitochondrial complex I (NDUFA6), AMPK, and MYL3—we conducted molecular docking studies. The docking results indicated that these active ingredients formed hydrogen bonds with key residues in the targets, with binding energies lower than − 7.0 kcal/mol (Fig. 9A), suggesting strong binding activity. We selected a pair of 1,5-dicaffeoylquinic acid and the NDUFA6 protein with the lowest binding energy for verification through CETSA and SPR experiments. The CETSA experiment revealed that treatment with 1,5-dicaffeoylquinic acid resulted in a significant shift in the melting curve of the NDUFA6 protein compared to the control group, particularly in the temperature range of 49,56 and 60 °C. These data demonstrate that treatment with 1,5-dicaffeoylquinic acid increased the thermal stability of the NDUFA6 protein, indicating its binding to the NDUFA6 protein (Fig. 9B, C). As shown in Fig. 9D, E, NDUFA6 bound to 1,5-dicaffeoylquinic acid with a dissociation constant (K_D_) of 7.35 × 10^–7^ M.

### 1,5-dicaffeoylquinic acid, verproside, catalposide alleviate IL-1β-induced chondrocyte injury by regulating the mitochondrial complex 1/AMPK/MYL3 pathway

**Fig. 10:**
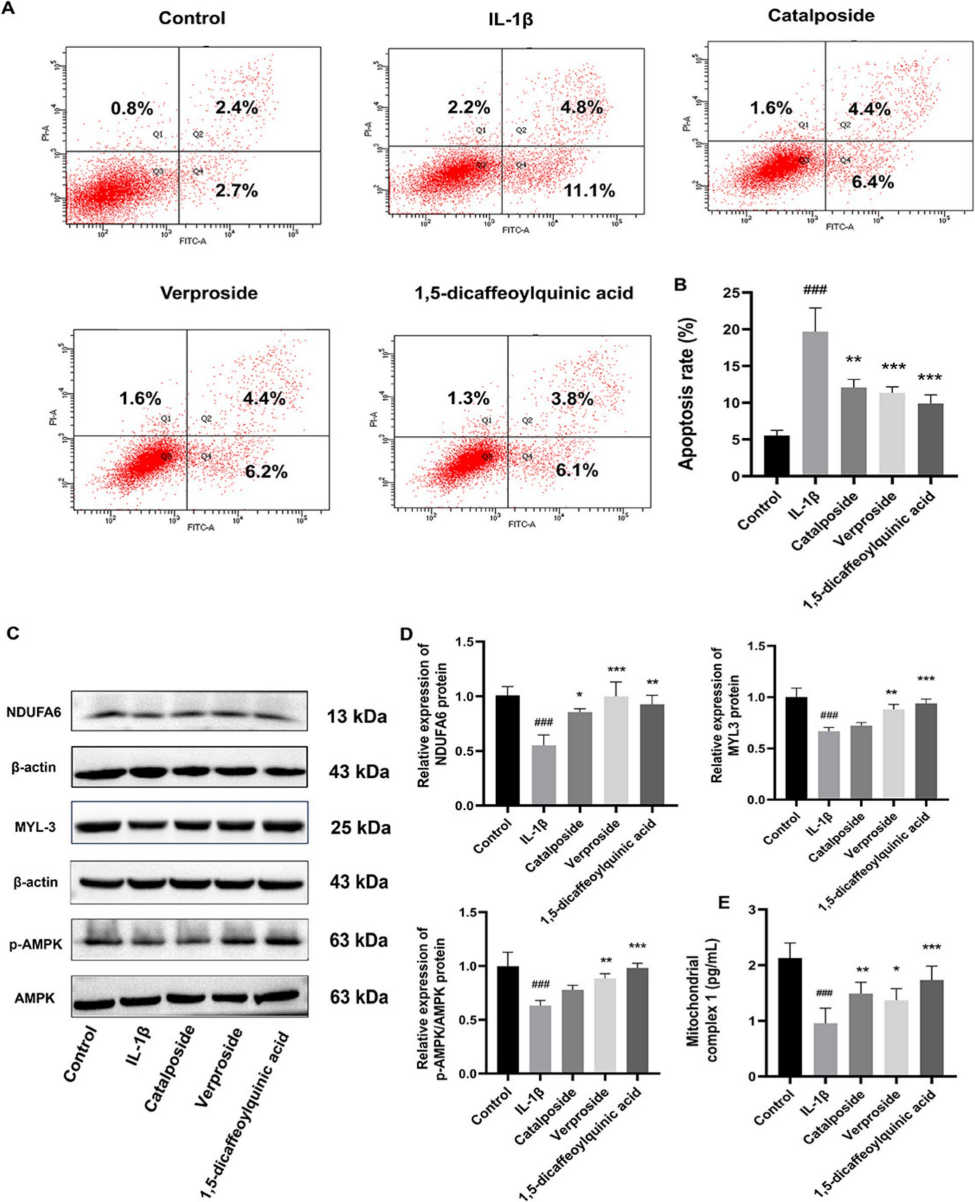
Mitigation of IL-1β-induced chondrocyte injury by 3 active ingredients. **A**, **B** Annexin V-FITC/PI flow cytometry was employed to detect apoptosis in chondrocytes across different groups (n = 3). **C**, **D** Western blot analysis assessed the protein expression of NDUFA6, MYL3, AMPK, p-AMPK, and their statistical results (n = 3). **E** Comparative analysis of mitochondrial respiratory chain complex I content in chondrocytes from various groups (n = 6). Data are expressed as mean ± SD, ^#^*P* < 0.05, ^##^*P* < 0.01, ^###^*P* < 0.001, comparison with the control group, *P < 0.05, **P < 0.01, ***P < 0.001, comparison with the IL-1β group

Compared to the control group, the apoptosis rate in the IL-1β group was significantly increased (*P* < 0.001). In contrast, the apoptosis rates in the catalposide, verproside, and 1,5-dicaffeoylquinic acid groups were significantly reduced (*P* < 0.01, *P* < 0.001, *P* < 0.001), as illustrated in Fig. 10A, B. Among the three components, verproside and 1,5-dicaffeoylquinic acid exhibited significant restorative effects on IL-1β-induced protein expression impairment. Specifically, both compounds significantly upregulated the expression of NDUFA6 (*P* < 0.001, *P* < 0.01) and MYL3 (*P* < 0.01, *P* < 0.001), while increasing the p-AMPK/AMPK ratio (*P* < 0.01, *P* < 0.001). In contrast, catalposide showed a significant upregulating effect on MYL3 protein expression (*P* < 0.05), as shown in Fig. 10C, D. Compared to the control group, the content of mitochondrial respiratory chain complex I in chondrocytes in the IL-1β group was significantly reduced (*P* < 0.001). Conversely, the content of mitochondrial respiratory chain complex I in the catalposide, verproside, and 1,5-dicaffeoylquinic acid groups was significantly increased (*P* < 0.01, *P* < 0.05, *P* < 0.001), as depicted in Fig. 10E.

## Discussion

KOA is a leading cause of disability among the elderly; however, no specific medications have been approved for its treatment [42]. The recently published"Chinese Guidelines for Diagnosis and Treatment of Osteoarthritis 2024"emphasize the use of reparative therapies in the early stages of KOA, including cartilage repair medications and joint injections [43]. Nevertheless, these options can be expensive and may have side effects, making it crucial to explore alternatives that alleviate pain and promote cartilage repair.

Natural medications have gained popularity due to their favorable bioactivity and reduced side effects compared to synthetic drugs. One such option is DAP, a Traditional Chinese Medicine transdermal patch made from two herbs, which has been clinically used for decades to treat arthritic conditions, including KOA. KOA is characterized by cartilage degeneration, bone sclerosis, and low-grade joint inflammation, leading to cartilage destruction [44]. The condition is often associated with an imbalance in the synthesis and degradation of the ECM, primarily composed of COL2A1, which is crucial for maintaining cartilage homeostasis[45]. MMP13 is a key enzyme involved in cartilage degradation [46]. Our study is the first to evaluate the therapeutic effects of DAP in a KOA rat model. We found that DAP increases COL2A1 levels, decreases MMP13, and alleviates cartilage degeneration. Additionally, we demonstrated that DAP can enhance the weight-bearing capacity and motor abilities of KOA rats. Notably, water transport within cartilage is crucial in the pathophysiology of KOA, especially in the early stages [4]. Dysregulation of aquaporins, such as AQP1, AQP3, and AQP4, has been linked to the development of KOA, contributing to matrix degradation and chondrocyte apoptosis [47]. Our findings suggest that DAP treatment effectively reduces joint swelling and the abnormal upregulation of aquaporin proteins.

Mitochondrial dysfunction and elevated levels of ROS are closely associated with KOA [48, 49]. The ETC generates ATP through redox reactions involving complexes I, II, III, and IV, with complexes I serving as primary sites for ROS formation [50]. In KOA, impaired electron transfer and disrupted energy metabolism lead to reduced ATP production and increased ROS levels, indicating mitochondrial dysfunction [51]. This mitochondrial impairment enhances the release of inflammatory mediators, including IL-1β, interleukin-6 (IL-6), and PGE2[52].Complex I is particularly significant in ROS generation; defects in this complex contribute to increased ROS levels and decreased antioxidant defenses [53, 54]. Key genes involved in the assembly and function of complex I include NDUFA6, NDUFS6, and NDUFA5[55–57]. NDUFA6 stabilizes the TMH1-2ND3 loop, which is essential for energy conversion [58], while NDUFS6 is crucial for complex I biogenesis; its deletion impairs ATP and superoxide production [39]. Although NDUFA5 is not directly involved in catalytic reactions, it facilitates electron transfer to the respiratory chain, thereby contributing to mitochondrial dysfunction [59]. Our studies have demonstrated that treatment with DAP markedly ameliorates the aberrant mRNA and protein expression levels of mitochondrial complex I components (NDUFA6, NDUFA5, NDUFS6), indicating that DAP may enhance mitochondrial function in chondrocytes and impede the progression of KOA. Compromised mitochondrial function has been shown to increase ROS production, subsequently diminishing AMPK activation [60]. AMPK is widely recognized as a crucial regulator of cellular energy homeostasis. Scientific investigations demonstrate that inhibited AMPK function induces both oxidative damage and inflammation within chondrocytes, consequently accelerating the degradation of the cartilaginous matrix [61]. Our data reveal that DAP treatment not only significantly reduces the levels of inflammatory cytokines and ROS but also enhances AMPK phosphorylation, thereby mitigating oxidative stress in chondrocytes and preventing cartilage matrix degradation. Furthermore, decreased expression of MYL3 in the articular cartilage of KOA, particularly within damaged cartilage, correlates with the progression of joint degeneration. This reduction in MYL3 activity exacerbates the action of enzymes responsible for chondrocyte ECM degradation, further facilitating cartilage deterioration and injury [41]. Our results indicate that DAP effectively increases MYL3 protein expression. Additionally, through Co-IP experiments, we have identified a link between AMPK and MYL3, highlighting a potential therapeutic target for reducing cartilage degeneration.

Exploring the skin-penetrating components of drugs is essential for advancing transdermal drug delivery systems in Traditional Chinese Medicine and for understanding their pharmacological properties. In our study, we utilized a Franz diffusion cell to analyze diffusion samples of DAP using UPLC-Q-TOF/MS, identifying 28 transdermal ingredients that penetrate the skin and exhibit anti-KOA properties, including iridoids, organic acids, flavonoids, caffeoylquinic acids, and carbohydrates. In vitro analyses revealed that 19 of these active ingredients displayed significant anti-inflammatory effects. Molecular docking studies on key transdermal ingredients—1,5-dicaffeoylquinic acid, catalposide, and verproside—demonstrated their ability to influence targets such as NDUFA6, AMPK, and MYL3, highlighting the synergistic action of DAP through multiple active ingredients and targets, further supported by CETSA and SPR findings. Subsequent experiments showed that 1,5-dicaffeoylquinic acid, verproside, and catalposide protect against IL-1β-induced chondrocyte damage by modulating the mitochondrial complex I (NDUFA6)/AMPK/MYL3 pathway. Notably, 1,5-dicaffeoylquinic acid inhibits MDA production, maintains catalase levels, enhances glutathione reductase activity, and reduces nitric oxide (NO), IL-6, and TNF-α levels, exhibiting both antioxidant and anti-inflammatory effects [62]. It also restores the GSH/ROS balance, mitigates mitochondrial depolarization, and alleviates mitochondrial dysfunction [63]. Its concentration in DAP is significantly higher than that of other ingredients, underscoring its pivotal role in KOA treatment. Furthermore, catalposide and verproside inhibit inflammatory mediators and downregulate matrix metalloproteinases via the NF-kB signaling pathway, thereby slowing cartilage degeneration [64]. Thus, 1,5-dicaffeoylquinic acid, catalposide, and verproside are crucial active ingredients in DAP for the treatment of KOA. In the subsequent research stage, relevant animal models should be utilized to supplement and validate the transdermal pharmacological mechanisms of representative active ingredients. This approach will enhance the robustness of experimental evidence supporting the transdermal efficacy of these representative agents.

## Conclusion

This study integrates chemical and transcriptomic analyses, network construction, and computational modeling, along with in vivo and in vitro validation, to identify potential bioactive ingredients contained in DAP. The findings suggest that DAP can ameliorate KOA partially modulating mitochondrial complex I/AMPK/MYL3 signaling, thereby providing protective effects on cartilage.These results offer experimental evidence that supports the scientific understanding and clinical application of DAP in the treatment of KOA.

## Supplementary Information

Additional file 1: Materials S1 Drugs. Methods S2 KOA animal model. Methods S3 Histological analysis. Methods S4 TUNEL staining. Methods S5 Preparation of DAP samples. Methods S6 Preparation of DAP in vitro transdermal sample. Methods S7 UPLC-Q-TOF/MS detection. Methods S8 Immunohistochemical staining. Methods S9 Cell viability assay. Methods S10 SPR assay.

Additional file 2: Table S1 DAP Dosage Scale. Table S2 Antibody information. Table S3 Gradient elution program. Table S4 Primer sequences for quantitative q-PCR. Table S5 Information on 19 main transdermal compound standards in the DAP. Table S6 Components detected by UHPLC-QTOF-MS in the positive and negative ion modes of DAP. Table S7 Identification results of transdermal components detected by UHPLC-QTOF-MS in the positive and negative ion modes of DAP.

Additional file 3: Fig. S1 A 206 reverse regulatory genes of DAP were screened and B-D GO enrichment analysis of the gene of anti-KOA effect of DAP. Fig. S2. Interaction between MYL3 protein and p-AMPK protein. A ZDOCK software predicts the interaction between AMPK and MYL3 proteins. (Purple area represents AMPK protein, blue area represents MYL3 protein, white circled area represents predicted binding region, yellow represents hydrogen bond. B-D Western blot was used to detect endogenous interaction between AMPK and MYL3 protein (n =3). Fig. S3. BPI chromatogram of DAP compounds. (A: negative ion mode; B: positive ion mode). Fig. S4. BPI chromatogram of DAP transdermal component negative ion mode. (A: Blank skin component; B: Transdermal component). Fig. S5. BPI chromatogram of DAP transdermal component positive ion mode. (A Blank skin component; B Transdermal component.) Fig. S6.CCK-8 was used to detect the changes in cell proliferation after 24 h treated with 19 compounds at different concentrations (0, 25, 50, 100 μM). Fig. S7. Top 60% of transdermal compounds with anti-inflammatory activities (n =6).

## Data Availability

The data in this study are available from the corresponding author upon reasonable request.

## Abbreviations

AHT: Artemisia halodendron Turcz. exBess
AMPK: Adenosine 5 ‘-monophosphate (AMP)-activated protein kinase
AQP1: Aquaporin 1
AQP3: Aquaporin 3;AQP4, Aquaporin 4
CETSA: Cellular thermal shift assay
ATP: Adenosine triphosphate
COL2A1: Collagen type II alpha 1
Co-IP: Co-Immunoprecipitation
DAP: Detumescence Analgesic Plaster
DCFH-DA: Dichlorodihydrofluorescein diacetate
DEGs: Differentially expressed genes
ECM: Extracellular matrix
ELISA: Enzyme-linked immunosorbent assay
ETC: Electron transport chain
GO: Gene Ontology
GSH: Glutathione
H&E: Hematoxylin and eosin
IHC: Immunohistochemical staining |
IL-1β: Interleukin-1beta
KEGG: KyotoEncyclopedia of Genes and Genomes
KOA: Knee osteoarthritis
MDA: Malondialdehyde|
MMP: Mitochondrial membrane potential
MMP13: Matrix metallopeptidase 13
MYL3: Myosin light chain 3
NDUFA5: NADH-ubiquinone oxidoreductase 1 alpha subcomplex 5
NDUFA6: NADH dehydrogenase (ubiquinone) 1 alpha subcomplex 6
NDUFS6: NADH-ubiquinone oxidoreductase Fe-S protein 6OA, Osteoarthritis
OXPHOS: Oxidativephosphorylation
PCA: Principal components analysis
PGE2: Prostaglandin E2
PPI: Protein–protein interaction network
qPCR: Quantitative real-time PCR
QZXTP: Qizheng Xiaotong Plaster
ROS: Reactive oxygen species
|RLU: Relative light units
RNA-seq: RNA-sequencing
SD: Sprague Dawley
SPF: Specific Pathogen Free
SPR: Surface plasmon resonance
SOD: Superoxide dismutase
TNF-α: Tumor necrosis factor-alpha
UPLC-Q-TOF/MS: Ultra Performance Liquid Chromatography-Quadrupole-Time-of-Flight Mass Spectrometry
VLP: Veronica linariifolia Pall. Ex Link subsp. Dilatate (Nakai et Kitagawa) Hong
