# Supplementary material for: Detumescence Analgesic Plaster mitigates knee osteoarthritis via active ingredients targeting mitochondrial complex 1/AMPK/MYL3-regulated cartilage homeostasis
Source: Chin Med. 2025 Oct 20;20:175. doi: 10.1186/s13020-025-01215-w (PMC12536539; doi:10.1186/s13020-025-01215-w)
Supplement: Supplementary file 1 — Additional file 1: Materials S1 Drugs. Methods S2 KOA animal model. Methods S3 Histological analysis. Methods S4 TUNEL staining. Methods S5 Preparation of DAP samples. Methods S6 Preparation of DAP in vitro transdermal sample. Methods S7 UPLC-Q-TOF/MS detection. Methods S8 Immunohistochemical staining. Methods S9 Cell viability assay. Methods S10 SPR assay. [file 13020_2025_1215_MOESM1_ESM.docx]

**Supplementary Materials for**

**Detumescence Analgesic Plaster mitigates knee osteoarthritis via active ingredients targeting mitochondrial complex 1/AMPK/MYL3-regulated cartilage homeostasis**

**Chunxia Li ^1^**^#^**, Weijie Li** **^1^**^#^**, Yue Yin ^1^, Xiaomei Xiang ^1^, Lu Fu ^1^, Ping Wang ^1^, Yanqiong Zhang ^1^**^*^**,** **Haiyu Xu ^12^**^*^

**^1^ *State Key Laboratory for Quality Ensurance and Sustainable Use of Dao-di Herbs, Institute of Chinese Materia Medica, China Academy of Chinese Medical Sciences, 100700, China***

***^2^College of pharmacy, Liaoning University of Traditional Chinese Medicine, Liaoning, China***

***Correspondence authors.**

***E-mail addresses:*** [**yqzhang@icmm.ac.cn**](mailto:yqzhang@icmm.ac.cn) **(Yanqiong Zhang), hyxu@icmm.ac.cn (Haiyu Xu).**

**^#^ ﻿These authors make equal contributions to this work.**

**Supplementary Materials and Methods**

**Materials S1 Drugs**

Extraction conditions and extraction solution processing of DAP

AHT: Grind the artemisia seeds into fine powder, add an appropriate amount of water and stir to form an ointment. Freeze dry and remove to obtain. Menthol: Take another menthol, add an appropriate amount of ethanol and stir to dissolve, seal and set aside for later use; VLP: adding 10 times the amount of water for the first time and boiling for 2 hours, and adding 8 times the amount of water for the second time and boiling for 1.5 hours. Combine the decoctions, filter, and concentrate the filtrate to a relative density of 1.10-1.12 of the extract. Cool to room temperature.

Qizheng Xiaotong Plaster (QZXTP), procured Tibet Cheezheng Tibetan Medicine Co., Ltd. (Xizang, China, Approval No. Z54020113) was employed as a positive control. It is worth noting that QZXTP was chosen as the positive drug for the following reasons: Firstly, as a prevalent Chinese external patch for KOA treatment, QZXTP is a Tibetan herbal formula known for its effects of promoting blood circulation, removing blood stasis, reducing swelling, and relieving pain, which are similar to the effects of DAP. Secondly, both QZXTP and DAP consist of medicinal solutions and patches, and both are external patches that can minimize interference with the differences observed in our experimental results.

**Methods S2 KOA animal model**

On the 40th day post-modeling (it should be noted that, considering the condition of the animals and the objective factors of the experiment, we conducted the sampling 2 days earlier), all rats were anesthetized via intraperitoneal injection of pentobarbital. Blood samples were collected and subjected to centrifugation to obtain serum samples, which were subsequently stored at -80 °C for further analysis. The right knee joints were surgically excised and fixed in 4% paraformaldehyde for histological analysis, while the remaining knee joint specimens were also stored at -80 °C for additional analysis.

**Methods S3 Histological analysis**

After fixation in 4% paraformaldehyde for 7 days, the samples were decalcified in 20% EDTA (pH 7.4), with the decalcification solution being replaced every 3 days until the tissue softened. The samples were then dehydrated prior to paraffin embedding. Sagittal slices, 5 μm thick, were prepared for H&E and Safranin O-fast green staining.

Histopathological assessment of synovitis. Tissue sections were stained with hematoxylin and eosin (H&E) to analyze synovitis (inflammatory cell influx and synovial hyperplasia) [1].

**Methods S4 TUNEL staining**

The cartilage tissues were fixed in 4% paraformaldehyde for 1 h and then incubated for 10 min each with 3% H_2_O_2_ and 0.2% Triton X-100. After 3 washes with PBS, the cells were stained using the TUNEL staining solution and DAPI.

**Methods S5**

**Preparation of DAP samples**

AHT: Grind the artemisia seeds into fine powder, add an appropriate amount of water and stir to form an ointment. Freeze dry and remove to obtain. Menthol: Take another menthol, add an appropriate amount of ethanol and stir to dissolve, seal and set aside for later use; VLP: adding 10 times the amount of water for the first time and boiling for 2 hours, and adding 8 times the amount of water for the second time and boiling for 1.5 hours. Combine the decoctions, filter, and concentrate the filtrate to a relative density of 1.10-1.12 (70C) of the extract. Cool to room temperature.

The DAP source is as mentioned above, Chlorogenic acid (purity: 98.76%), caffeic acid (purity: 99.30%), 1,5-dicaffeoylquinic acid (purity: 98.42%) were purchased from Shanghai Taozhu Biotechnology Co., Ltd (Shanghai, China). Preparation method of DAP according to the process: Mix 1g of AHT 0.08 g of VLP, and 0.004 g of menthol, freeze-dried powder raw materials, and place them in a 100 mL conical flask. Add 10 mL of 50% methanol, shake well, weigh, sonicate for 30 minutes, add ice cubes to water, cool down, and make up for the weight loss. Centrifuge at high speed and low temperature of 12000r/min for 10 minutes, and pass through 0.22 μm microporous filter membrane, take the filtrate and obtain it.

**Methods S6 Preparation of** **DAP in vitro transdermal sample**

Using the Franz diffusion cell method, the effective area of the diffusion cell is 3.14 cm^2^, and the volume of the receiving cell is 10 mL. The skin used in the experiment was the abdominal skin of male SD rats (4 weeks old, 120 ±10g). rats, which was prepared by removing the fat layer, fascia, and adhesion, and washing with PBS (pH=7.4). Take an appropriate size of rats abdominal skin and clip it between the supply and receiving pools, ensuring that its stratum corneum faces the supply pool. The liquid surface is in complete contact with the inner layer of the skin. After installing the device, place the prepared mixture of anti-inflammatory and analgesic raw materials in the supply tank. The receiving liquid is 30% ethanol, the magnetic stirring speed is 350 r/min, and the temperature is controlled at (32+0.2) °C. After balancing for 0.5 h, at the time point of 24 h, all 9 mL of the receiving solution will be taken out, and at the same time, 1 mL of new receiving solution of the same volume will be added. Take 9 mL of the reception solution, put it into a 10 mL centrifuge tube, dry it with nitrogen, dissolve it in 0.2 mL of 25% ethanol, centrifuge at high speed and low temperature of 12000 r/min for 10 minutes, pass it through a 0.22 μm microporous filter membrane, and take the filtrate to obtain it.

**Methods S7 UPLC-Q-TOF/MS detection**

The samples were analyzed using the Waters ACQUITY UPLC system (Waters, Milford, Pennsylvania, USA) and Waters Q-TOF advanced mass spectrometer. All analytes are in ACQUITY UPLC HSS T3, separate on the 1.8 μm (2.1x100mm) column. The mobile phase consists of 0.1% formic acid aqueous solution solvent A and 0.1% formic acid acetonitrile solvent B, with a flow rate of 0.5 mL/min; Injection volume: 2 μL; Column temperature: 30 °C, gradient elution was described in **Table gradient elution program.** The data analysis was conducted using Masslynx 4.1 software combined with Waters UNIF TM 1.9.4 software for data collection and analysis. The collected data format was Continuous data.

The electrospray ion source (ESI) is used, and the scanning mode is full information tandem mass spectrometry (MSE) mode. In the positive and negative ion modes, the operating parameters are capillary voltage 3.0 kV, taper hole voltage 30 V, taper hole backblowing gas flow (N2) 50 L/h, desolvent gas flow 1000 L/h, ion source temperature 120 ℃, desolvent gas temperature 450 ℃, scanning range m/z 50~1500, low collision energy 20 eV, high collision energy 70 eV. During the process, leucine enkephalin (m/z 556.2771 ESI+, m/z 554.2615 ESI -) is used to calibrate the mass axis to ensure the accuracy of mass spectrometry data. The data analysis was conducted using Masslynx 4.1 software combined with Waters UNIFITM 1.9.4 software for data collection and analysis, with the collected data format being Continuum data.

Table 1-3 Gradient elution procedure

| Time（min） | B乙腈（v%） |
| --- | --- |
| 0 | 5% |
| 10 | 28% |
| 10.2 | 38% |
| 12 | 55% |
| 13 | 95% |
| 13.2 | 5% |
| 16 | 5% |

**Methods S8 Immunohistochemical staining (IHC)**

Antigen retrieval was performed by boiling specimens in sodium citrate buffer. After blocking with BSA for 1 h, the specimens were incubated overnight in a humidified chamber at 4 °C HRP-conjugated secondary antibody was applied to detect the primary antibody for 30 min on the second day. Subsequently, the DAB substrate was utilized for coloration. The sample was then examined under a white light microscope for result interpretation. The nucleus, stained with hematoxylin, appeared blue, whereas the positive expression of DAB exhibited a brownish-yellow color.

**Methods S9 Cell viability assays**

The cells achieved complete attachment to the wall within 24 h. Following stimulation with 19 transdermal compounds at varying concentrations (0, 25, 50, 100 μM) for 24 h, the previous medium was discarded. Subsequently, 100 μL of 10% CCK-8 solution was added to each cell well (with medium dilution), ensuring that no bubbles were formed during the procedure. The cells were incubated at 37 °C for 1.5 h, after which the OD values were measured at 450 nm using an enzyme-linked instrument. Three independent experiments were performed to ensure the reliability of the results.

**Methods S10 SPR**

Firstly, the NDUFA6 Protein (D4A3V2-M1-P130, MCE, USA) was immobilized on the hannel 2 of CM5 chip (BR100530, Cytiva, Sweden) after being diluted to 50 μg/ml in the sodium acetate buffer (pH4.0). The 1,5-dicaffeoylquinic acid (DSTDE000602, Chengdu Desire Biotechnology, China) was diluted to different concentrations of 0.03125μM, 0.0625μM, 0.125μM, 0.25μM, 0.5 μM, and 1μM at a flow rate of 30 μl/min, and duration is 150 s. Finally, glycine HCl (10mM, pH2.0) was used to renew the chip surface.

References

[1] Barbosa GM, Cunha JE, Cunha TM, Martinho LB, Castro PATS, Oliveira FFB, et al. Clinical-like cryotherapy improves footprint patterns and reduces synovial inflammation in a rat model of post-traumatic knee osteoarthritis. Sci Rep. 2019; 10; 9 (1):14518.
