# Supplementary material for: Detumescence Analgesic Plaster mitigates knee osteoarthritis via active ingredients targeting mitochondrial complex 1/AMPK/MYL3-regulated cartilage homeostasis
Source: Chin Med. 2025 Oct 20;20:175. doi: 10.1186/s13020-025-01215-w (PMC12536539; doi:10.1186/s13020-025-01215-w)
Supplement: Supplementary file 2 — Additional file 2: Table S1 DAP Dosage Scale. Table S2 Antibody information. Table S3 Gradient elution program. Table S4 Primer sequences for quantitative q-PCR. Table S5 Information on 19 main transdermal compound standards in the DAP. Table S6 Components detected by UHPLC-QTOF-MS in the positive and negative ion modes of DAP. Table S7 Identification results of transdermal components detected by UHPLC-QTOF-MS in the positive and negative ion modes of DAP. [file 13020_2025_1215_MOESM2_ESM.docx]

**Supplementary Table 1-7**

**Table S1** DAP Dosage Scale

| Clinical equivalent dose | | Dose (g/kg) | Administration weight per rat (g) | Required volume of medication (mL) |
| --- | --- | --- | --- | --- |
| 1 times | 0.0367 | | 0.00734 | 0.0352 |
| 2 times | 0.0735 | | 0.0147 | 0.0705 |
| 4 times | 0.147 | | 0.0294 | 0.141 |

**Table S2** Antibody information

| Abbreviation of antibody name | Full name of antibody | manufacturer | Item number | purpose |
| --- | --- | --- | --- | --- |
| MMP13 | Matrix metalloprotease 13 | Proteintech | 18165-1-AP | WB |
| COL2A1 | Collagen type II alpha-1 gene | Proteintech | 28459-1-AP | WB |
| MYL3 | Myosin light chain 3 | Proteintech | 10913-1-AP | WB/IHC |
| NDUFA5 | NADH-ubiquinone oxidoreductase 1 alpha subcomplex 5 | Proteintech | 16640-1-AP | WB/IHC |
| NDUFA6 | NADH dehydrogenase [ubiquinone] 1 alpha subcomplex subunit 6 | Proteintech | 15445-1-AP | WB/IHC |
| NDUFS6 | NADH dehydrogenase (ubiquinone) 1 alpha subcomplex 6 | Proteintech | 14417-1-AP | IHC |
| AMPK | Adenosine 5‘-monophosphate (AMP)-activated protein kinase | immunoway | YT0216 | WB/IHC |
| p-AMPK | phosphorylation-AMP-activated protein kinase | immunoway | YP0575 | WB/IHC |
| GAPDH | Glyceraldehyde-3-phosphate dehydrogenase | abcam | ab8245 | WB |

**Table S3 Gradient elution program**

| Time（min） | B Acetonitrile（v%） |
| --- | --- |
| 0 | 5% |
| 10 | 28% |
| 10.2 | 38% |
| 12 | 55% |
| 13 | 95% |
| 13.2 | 5% |
| 16 | 5% |

**Table S4** Primer sequences for quantitative RT-PCR.

| **Gens** | **Primer sequences** |  |
| --- | --- | --- |
| GAPDH | Forward  Reverse | GGCAAGTTCAACGGCACAGTC TCGCTCCTGGAAGATGGTGATG |
| MYL3 | Forward  Reverse | GACAAGGAGGGCAACGGAACTG  TCGTCTTCTGTCAGCCTCTCACC |
| Ndufa5 | Forward | TGCGACACTCCACACGAGAGG |
|  | Reverse | TCTGTAGGCTGCATGTTTAGGAAAGTG |
| Ndufa6 | Forward | CGGGAGGTGCCAAACACTGTG |
|  | Reverse | GGTCGGTGACATGGGCATTCTTC |
| Ndufs6 | Forward | AGGAGGATTCGTTTTGTAGATCGTCAG |
|  | Reverse | GATACGATGGTCCACCTCATTCACAG |
| Cox6a2 | Forward | TGCTCGCTTAACTGCTGGATGC |
|  | Reverse | GAGAAGGGCTTGGTTCGGATGC |

**Table S5** Information on 19 main transdermal compound standards in the DAP

| English name | Molecular weight | CAS | Companys |
| --- | --- | --- | --- |
| Citric acid | 192.12 | 77-92-9 | Shanghai Taozhu Biotechnology Co., Ltd |
| Protocatechuic acid | 154.12 | 99-50-3 | - |
| 4-Caffeoylquinic acid | 354.31 | 905-99-7 | - |
| Geniposidic Acid | 374.34 | 27741-01-1 | - |
| Chlorogenic Acid | 354.31 | 327-97-9 | - |
| Ferulic Acid | 194.18 | 1135-24-6 | - |
| Caffeic Acid | 180.16 | 331-39-5 | - |
| Rutin | 153-18-4 | 153-18-4 | - |
| Catalposide | 482.43 | 6736-85-2 | - |
| Picroside II | 512.46 | 39012-20-9 | - |
| Plantamajoside | 640.59 | 104777-68-6 | - |
| Picroside I | 492.47 | 27409-30-9 | - |
| Apigenin 7-glucoside | 432.38 | 578-74-5 | - |
| p-Hydroxybenzaldehyde | 122.12 | 123-08-0 | - |
| 3-Indoleacrylic acid | 187.19 | 1204-06-4 | - |
| Umbelliferone | 162.14 | 93-35-6 | - |
| 1,5-dicaffeoylquinic acid | 516.45 | 30964-13-7 | Chengdu Desite Biotechnology Co., Ltd |
| Verproside | 498.43 | [50932-20-2](https://www.chemsrc.com/baike/1555952.html) | - |
| 6-Feruloylcatalpol | 538.5 | 770721-33-0 | - |

**Table S6** Identification results of components detected by UHPLC-QTOF-MS/MS in the positive and negative ion mode of DAP

| NO. | TR (min) | Observed  M/Z | Theoretical  M/Z | Mass  error | Response | Adducts | Formulas | fragmentation patterns | | Potential compounds | | Herbs | |
| --- | --- | --- | --- | --- | --- | --- | --- | --- | --- | --- | --- | --- | --- |
| 1 | 0.58 | 195.0513 | 150.0528 | 0.24 | 122816 | +HCOO | C_5_H_10_O_5_ | | - | | DL-Arabinose | | SHZ |
| 2 | 0.6 | 341.1092 | 341.1084 | 0.22 | 60615 | -H | C_12_H_22_O_11_ | | M/Z195.0522[M-H-C_6_H_5_O_5_]^-^  M/Z 179.0567[M-H-C_6_H_6_O_6_]^-^ | | Sucrose | | SHZ |
| 3 | 0.6 | 381.0792 | 381.0821 | 3.80 | 37200 | +H | C_17_H_16_O_10_ | | M/Z 365.1018 [M+H-O]^+^ | | 8-O-beta-D-Glucopyranosyl-5-hydroxypsoralen^b^ | | SHZ |
| 4 | 0.63 | 179.0567 | 179.0556 | 0.65 | 6540 | -H | C_6_H_12_O_6_ | | M/Z129.0203 [M-H-CH_6_O_2_]^-^ | | D-Galactose | | SHZ |
| 5 | 0.63 | 244.0919 | 244.0933 | 2.86 | 35300 | +H | C_9_H_13_N_3_O_5_ | | M/Z 112.0522[M+H-C_5_H_8_O_4_]^+^  M/Z 95.0243[M+H-C_5_H_8_O_4_-NH]^+^ | | Cytidine^b^ | | SHZ |
| 6 | 0.68 | 152.0558 | 152.0572 | 4.65 | 18800 | +H | C_5_H_5_N_5_O | | M/Z135.0292[M+H-N_2_]^+^ | | Guanine^b^ | | SHZ |
| 7 | 0.82 | 191.0208 | 191.0192 | -4.18 | 393927 | -H | C_6_H_8_O_7_ | | M/Z173.0133[M-H-H_2_O]^-^M/Z129.0203[M-H-H_2_O^-^C_2_O]^-^、M/Z111.0098[M-H^-^H_2_O-C_2_O-H_2_O]^-^ | | Citric acid | | SMJ |
| 8 | 0.88 | 153.0427 | 153.0412 | -4.96 | 10800 | +H | C_5_H_4_N_4_O_2_ | | M/Z136.0784[M+H-N_2_O]^+^ | | 2,6-Dihydroxypurine^b^ | | SHZ |
| 9 | 0.95 | 407.1196 | 362.1213 | 0.17 | 11073 | +HCOO | C_15_H_22_O_10_ | | M/Z199.0263[M-H-Glc]-m/z 181.9093[M-H-Glc-H_2_O]^-^ | | Catalpol | | SMJ |
| 10 | 0.95 | 276.1419 | - | - | 10700 | - | - | | - | | Unknown | | SHZ |
| 11 | 0.97 | 123.0445 | 123.0446 | 1.26 | 1163 | +H | C_7_H_6_O_2_ | | M/Z119.0517[M+H+CH_2_-O_2_]^+^ | | 4-Hydroxybenzaldehyde | | SHZ |
| 12 | 1.00 | 268.1055 | 268.1046 | -1.67 | 38700 | +H | C_10_H_13_N_5_O_4_ | | M/Z136.0619[M+H-C_5_H_8_O_4_]^+^  M/Z119.0363[M+H-C_5_H_8_O_4_-HN]^+^ | | Adenosin^b^ | | SHZ |
| 13 | 1.07 | 136.062 | 136.0623 | 1.15 | 10600 | +H | C_5_H_5_N_5_ | | M/Z119.0363[M+H-HN]^+^ | | Adenine^b^ | | SHZ |
| 14 | 1.09 | 391.1257 | 346.1214 | 0.26 | 6006 | +HCOO | C_15_H_22_O_9_ | | M/Z276.1070 [M+COOH^-^C_3_H_2_O_2_]^-^ | | Aucubin | | SMJ |
| 15 | 1.12 | 152.0588 | - | - | 13400 | +H | - | | - | | Unknown | | SHZ |
| 16 | 1.17 | 388.2176 | - | - | 5840 | +H | - | | - | | Unknown | | SHZ |
| 17 | 1.22 | 152.0588 | - | - | 3380 | +H | - | | - | | Unknown | | SHZ |
| 18 | 1.4 | 283.0665 | 283.0679 | 2.47 | 51800 | -H | C_10_H_12_N_4_O_6_ | | M/Z151.0252[M-H-C5H8O4]^-^ | | Xanthosine^b^ | | SHZ |
| 19 | 1.4 | 285.0834 | 285.081 | -4.23 | 68265 | +H | C_16_H_12_O_5_ | | M/Z268.1055 [M+H-O]^+^ | | Genkwanin | | SHZ |
| 20 | 1.64 | 149.0626 | 149.0618 | 2.19 | 698 | +H | C_9_H_8_O_2_ | | M/Z119.0717[M+H-C_3_H_2_O_2_]^+^ | | Cinnamic acid | | SMJ |
| 21 | 1.77 | 219.1364 | 219.1385 | 4.79 | 3940 | +H | C_14_H_18_O_2_ | | M/Z 205.0710[M+H-H_2_O]^+^ | | Amyl cinnamate^b^ | | SHZ |
| 22 | 1.79 | 295.1292 | 295.1302 | -2.75 | 1104 | +H | C_19_H_18_O_3_ | | M/Z187.0652[M+H-C_5_O_3_]^+^ | | Tanshinone II A | | SHZ |
| 23 | 1.87 | 242.1489 | 243.1497 | 0.56 | 4540 | +H | C_15_H_18_N_2_O | | M/Z229.1223 [M+H-CH_2_]^+^ | | Huperzine A^b^ | | SHZ |
| 24 | 1.92 | 303.1043 | 303.1078 | 0.34 | 405 | +H | C_13_H_18_O_8_ | | M/Z158.0739 [M+H-C_5_H_5_O_5_]^+^ | | Tachioside | | SMJ |
| 25 | 2.09 | 243.1359 | 243.1385 | 5.34 | 2820 | +H | C_16_H_18_O_2_ | | M/Z187.0652 [M+H-C_2_O_2_]^+^ | | 1,3-dimethoxy-5-(2-phenylethyl) benzene^b^ | | SHZ |
| 26 | 2.1 | 167.0355 | 167.0345 | 0.54 | 6255 | -H | C_8_H_8_O_4_ | | M/Z 153.0180 [M-H-CH_2_]^-^ | | 3-hydroxy-4-methoxybenzoic acid | | SMJ |
| 27 | 2.14 | 255.0503 | - | - | 92400 | -H | - | | - | | Unknown | | SHZ |
| 28 | 2.29 | 255.0520 | - | - | 2870 | +H | - | | - | | Unknown | | SMJ |
| 29 | 2.41 | 153.0180 | 153.0188 | 0.64 | 72233 | -H | C_7_H_6_O_4_ | | M/Z109.03[M-H-CO_2_]^-^ | | Protocatechuic acid | | SMJ |
| 30 | 2.41 | 231.173 | 231.1749 | 4.12 | 4300 | +H | C_16_H_22_O | | M/Z153.0166[M+H-C_5_H_2_O]^+^ | | Cycloisolongifolene,8,9-dehydro-9-formyln^b^ | | SHZ |
| 31 | 2.63 | 389.1086 | 344.1107 | 1.79 | 6560 | +HCOO | C_15_H_20_O_9_ | | M/Z165.0549[M+COOH-C_2_H_10_O_5_-]^-^ | | Syringic acid-4-O-α-L-rhamnopyranoside^b^ | | SMJ |
| 32 | 2.74 | 353.0852 | 353.0873 | 2.97 | 8840 | -H | C_16_H_17_O_9_ | | M/Z 191.0549[M-H-C_9_H_6_O_3_]^-^ | | 4-Caffeoylquinic acid | | SHZ |
| 33 | 2.77 | 188.0728 | 188.0711 | -4.52 | 54800 | +H | C_11_H_9_NO_2_ | | M/Z146.0608[M+H-CHO_2_]^+^  M/Z118.0667[M+H-CHO_2_-C_2_H_4_]^+^ | | 3-Indoleacrylic acid^b^ | | SHZ |
| 34 | 3.08 | 253.6373 | - | - | 3350 | +H | - | | - | | Unknown | | SHZ |
| 35 | 3.11 | 373.1127 | 373.1135 | -0.47 | 23987 | -H | C_16_H_22_O_10_ | | M/Z193.0508[M-H-C_9_H_8_O_4_]^-^  M/Z149.0583[M-H-C_9_H_8_O_4_-C_2_H_4_O]^-^ | | Geniposidic  acid | | SMJ |
| 36 | 3.15 | 637.1796 | 637.1769 | -2.11 | 7250 | -H | C_29_H_34_O_16_ | | M/Z 499.1431 [M-H-C_6_HO_4_]^-^ | | Ombuoside^b^ | | SHZ |
| 37 | 3.29 | 265.1568 | 265.1592 | 4.45 | 16745 | +H | C_19_H_20_O | | M/Z163.0426 [M+H-C_8_H_4_]^+^ | | 3-phenanthrenol,7,8-dimethyl-2-(1-methylethyl)- ^b^ | | SHZ |
| 38 | 3.39 | 457.1346 | 457.1346 | 0.62 | 6920 | -H | C_20_H_26_O_12_ | | 353.0852 [M-H-C_4_H_8_O_3_]^-^ | | Regaloside E^b^ | | SHZ |
| 39 | 3.50 | 575.2349 | - | - | 3530 | -H | - | | - | | Unknown | | SHZ |
| 40 | 3.65 | 451.1312 | - | - | 2230 | -H | - | | - | | Unknown | | SHZ |
| 41 | 3.65 | 291.0975 | 291.1021 | 7.94 | 1980 | +H | C_19_H_14_O_3_ | | M/Z172.0775 [M+H-C_4_H_7_O_4_]^+^ | | Aurin^b^ | | SHZ |
| 42 | 3.78 | 295.1655 | 295.1698 | 7.23 | 3440 | +H | C_20_H_22_O_2_ | | M/Z169.0800 [M+H- C_6_H_8_O_3_]^+^ | | Eryvariestyrene^b^ | | SHZ |
| 43 | 3.79 | 515.1376 | 515.1401 | 2.42 | 29700 | -H | C_22_H_28_O_14_ | | M/Z 457.104[M-H-CO_3_]^-^ | | 5-O-(3'-O-Glucosylcaffeoyl) Quinic Acid^b^ | | SMJ |
| 44 | 3.81 | 583.1749 | 538.1686 | 8.15 | 18311 | +HCOO | C_25_H_30_O_13_ | | M/Z 375.0742 [M-H-C_6_H_10_O_5_]^-^ | | 6-Feruloylcatalpol | | SMJ |
| 45 | 3.88 | 707.1803 | 707.1824 | 1.48 | 126000 | 2M-H | C_32_H_36_O_18_ | | M/Z191.0549[M-H-C_9_H_6_O_3_]-  M/Z179.0549[M-H-C_7_H_10_O_5_]^-^  M/Z135.0549[M-H-C_8_H_10_O_5_]^-^ | | Chlorogenic acid^a b^ | | SHZ |
| 46 | 3.88 | 163.0381 | 163.0395 | -4.26 | 248324 | +H | C_9_H_6_O_3_ | | M/Z 135.0455 [M+H-C_2_H_4_]^+^ | | 7-Hydroxycoumarin^b^ | | SHZ |
| 47 | 4.01 | 239.0567 | 194.0579 | 2.56 | 246564 | +HCOO | C_10_H_10_O_4_ | | M/Z179.0332[M-H-CO_2_]- | | Ferulate | | SMJ |
| 48 | 4.13 | 305.1117 | - | - | 2880 | +H | - | | - | | Unknown | | SHZ |
| 49 | 4.29 | 619.1667 | 619.1663 | -3.23 | 8330 | -H | C_29_H_32_O_15_ | | M/Z457.1120[M-H-C_6_H_10_O_5_]-  M/Z457.1120[M-H-C_6_H_10_O_5_-CO]^-^ | | Camellianin A^b^ | | SHZ |
| 50 | 4.42 | 163.0381 | 163.0395 | 4.29 | 3170 | +H | C_9_H_6_O_3_ | | M/Z 135.0455 [M+H-C_2_H_4_]^+^ | | 3-Hydroxycoumarin^b^ | | SHZ |
| 51 | 4.43 | 179.0332 | 179.0345 | 3.63 | 64100 | -H | C_9_H_8_O_4_ | | M/Z135.0447[M-H-CO_2_]^-^ | | Caffeic acid^a^ | | SHZ |
| 52 | 4.65 | 499.1431 | 499.1452 | 2.15 | 10400 | -H | C_22_H_28_O_13_ | | M/Z 337.0794[M-H-C_6_H_10_O_5_]^-^ | | 5^-^ Methylcoumarin-4-cellobioside^b^ | | SMJ |
| 53 | 4.79 | 459.1319 | 459.1291 | -3.04 | 9550 | -H | C_23_H_24_O_10_ | | M/Z191.0549[M-H-C_13_H_16_O_6_]^-^ | | Afromosin 7-glucoside | | SHZ |
| 54 | 4.95 | 381.1753 | - | - | 1940 | -H | - | | - | | Unknown | | SHZ |
| 55 | 5.03 | 529.1543 | 529.1558 | 1.41 | 15000 | -H | C_23_H_30_O_14_ | | M/Z 167.0332[M-H-C_15_H_22_O_10_]^-^ | | Pikuroside^b^ | | SMJ |
| 56 | 5.03 | 351.1071 | - | - | 1170 | +H | - | | - | | Unknown | | SMJ |
| 57 | 5.23 | 541.1533 | - | - |  | -H | - | | - | | Unknown | | SMJ |
| 58 | 5.38 | 371.0953 | - | - | 15800 | -H | - | | - | | Unknown | | SMJ |
| 59 | 5.49 | 319.0842 | 319.0826 | 1.43 | 458 | +H | C_16_H_14_O_7_ | | M/Z305.1178 [M+H-C]^+^、M/Z273.0852 [M+H-C-CO_2_]^+^ | | Padmatin | | SMJ |
| 60 | 5.54 | 465.1501 | - | - | 26500 | -H |  | | - | | Unknown | | SHZ |
| 61 | 5.54 | 273.0852 | 273.0888 | -8.13 | 385 | +H | C_12_H_16_O_7_ | | M/Z172.0775 [M+H-C_4_H_5_O_4_]^+^ | | Arbutin | | SMJ |
| 62 | 5.61 | 497.1286 | 497.1295 | -1.55 | 620058 | -H | C_22_H_26_O_13_ | | M/Z335.0782[M-H-C_6_H_10_O_5_]^-^  M/Z221.0449[M-H-C_6_H_10_O_5_-C_4_H_2_O_4_]^-^M/Z153.0180[M-H-C_6_H_10_O_5_-C_9_HO_4_]^-^M/Z109.0300[M-H-C_6_H_10_O_5_-C_9_HO_4_-CO_2_]^-^ | | Verproside | | SMJ |
| 63 | 5.61 | 521.1271 | - | - | 1260 | +H | - | | - | | Unknown | | SMJ |
| 64 | 5.69 | 279.1685 | - | - | 2420 | +H | - | | - | | Unknown | | SHZ |
| 65 | 5.8 | 627.1546 | 627.1561 | -1.23 | 142390 | +H | C_27_H_30_O_17_ | | M/Z303.0492 [M+H-C_12_H_20_O_10_]^+^ | | Quercetin-3-sophoroside^b^ | | SMJ |
| 66 | 5.81 | 625.1393 | 625.1405 | 0.88 | 291244 | -H | C_27_H_30_O_17_ | | M/Z301.0366[M-H-2GIc]^-^ | | Quercetin-3-sophoroside^b^ | | SMJ |
| 67 | 6.1 | 381.189 | 381.1914 | 3.14 | 5060 | -H | C_20_H_30_O_7_ | | M/Z337.2022[M-H-C_2_H_4_O]^-^ | | Cinnacasol^b^ | | SHZ |
| 68 | 6.12 | 295.1897 | 295.1909 | 2.03 | 2370 | +H | C_17_H_26_O_4_ | | M/Z179.0595 [M+H-C_5_H_6_O_3_]^+^ | | Embelin^b^ | | SHZ |
| 69 | 6.12 | 151.1153 | 151.1134 | 1.75 | 2285 | +H | C_10_H_14_O | | M/Z121.0341 [M+H-CH_2_O]^+^ | | Phenol，2-methyl-5-(1-methylethyl) | | SHZ |
| 70 | 6.25 | 751.2114 | 751.2086 | -1.86 | 14300 | -H | C_34_H_40_O_19_ | | M/Z667.1503 [M-H-CH_8_O_4_]^-^ | | Arillatose A^b^ | | SHZ |
| 71 | 6.27 | 237.0761 | 237.0786 | 2.88 | 355 | +H | C_12_H_12_O_5_ | | M/Z 177.0585 [M+H-CO_3_]^+^ | | 6,7,8-Trimethoxycoumarin^b^ | | SHZ |
| 72 | 6.29 | 213.1592 | - | - | 2194 | +H | - | | - | | Unknown | | SMJ |
| 73 | 6.36 | 533.1064 | - | - | 2250 | -H | - | | - | | Unknown | | SHZ |
| 74 | 6.62 | 611.161 | 611.1612 | 0.25 | 29125 | +H | C_27_H_30_O_16_ | | M/Z 287.0576[M+H-C_12_H_20_O_10_]^+^ | | Rutin^b^ | | SMJ |
| 75 | 6.63 | 609.1454 | 609.1456 | 0.16 | 77872 | -H | C_27_H_30_O_16_ | | M/Z285.0428[M-H-C_12_H_20_O_10_]^-^M/Z193.0508[M-H-C_12_H_20_O_10_-C_6_H_4_O]- | | Rutin ^b^ | | SMJ |
| 76 | 6.83 | 284.1309 | 284.1286 | -4.05 | 1160 | +H | C_17_H_17_NO_3_ | | M/Z [M+H -NO_2_-C_2_H_3_]^+^ | | N-p-trans-Coumaroyltyramine^b^ | | SHZ |
| 77 | 6.84 | 481.1351 | 481.1346 | -0.76 | 343175 | -H | C_22_H_26_O_12_ | | M/Z319.0816[M-H-C_6_H_10_O_5_]^-^ | | Catalposide^a^ | | SMJ |
| 78 | 6.9 | 331.1752 | 331.1757 | 0.7.5 | 533 | +H | C_16_H_26_O_7_ | | M/Z 318.0727 [M+H-CH_2_]^+^ | | Picrocrocin^b^ | | SMJ |
| 79 | 7.08 | 669.1621 | 669.1608 | -0.97 | 17200 | +H | C_36_H_28_O_13_ | | M/Z 303.0492 [M+H-2GIc-C_4_H_2_O_2_]^+^ | | Bijaponicaxanthone^b^ | | SMJ |
| 80 | 7.08 | 303.0492 | 303.0503 | 0.35 | 2384 | +H | C_15_H_10_O_7_ | | M/Z 273.0445 [M+H-CH_2_O]^+^ | | Quercetin | | SMJ |
| 81 | 7.09 | 667.1503 | 667.1452 | -3.822 | 35700 | -H | C_34_H_30_O_15_ | | M/Z301.0366[M-H-2GIc-CH_6_O_4_]^-^ | | Bijaponicaxanthone^b^ | | SMJ |
| 82 | 7.1 | 179.1066 | 179.1081 | 1.40 | 1251 | +H | C_11_H_14_O_2_ | | M/Z 143.0433 [M+H-H_4_O_2_]^+^ | | Methyleugenol | | SHZ |
| 83 | 7.16 | 511.1443 | 511.1452 | -1.44 | 400493 | -H | C_23_H_28_O_13_ | | M/Z349.0933[M-H-GIc]^-^M/Z235.0609[M-H-GIc-C_5_H_6_O_3_]^-^ | | Picroside II | | SMJ |
| 84 | 7.16 | 513.1622 | 513.1603 | 0.44 | 893 | +H | C_23_H_28_O_13_ | | M/Z 495.1604 [M+H-H_2_O]^+^  M/Z 351.0418 [M+H- GIc]^+^ | | Picroside II | | SMJ |
| 85 | 7.17 | 523.1444 | 523.1452 | -1.33 | 209521 | -H | C_24_H_28_O_13_ | | M/Z349.0933[M-H-GIc]、  M/Z235.0609[M-H- GIc -C_5_H_6_O_3_]^-^  Z167.0332.[M-H-GIc- C_5_H_6_O_3_-CO-C_7_O_4_H_5_]^-^M/Z123.0473[M-H-GIc- C_5_H_6_O_3_-CO-CO_2_]^-^ | | Verminoside | | SMJ |
| 86 | 7.17 | 165.0580 | 165.0566 | 2.00 | 509 | +H | C_9_H_8_O_3_ | | M/Z 151.0418[M+H-O]^+^ | | P-coumaricacid | | SMJ |
| 87 | 7.25 | 533.1064 | - | - | 14000 | -H | - | | - | | Unknown | | SHZ |
| 88 | 7.34 | 473.2004 | - | - | 620 | - | - | | - | | Unknown | | SMJ |
| 89 | 7.42 | 511.1412 | 511.1393 | -1.85 | 12000 | -H | C_30_H_24_O_8_ | | M/Z235.0609 [M-H-GIc- C_5_H_10_O_4_]^-^ | | Gnetuhainin F^b^ | | SMJ |
| 90 | 7.58 | 477.1382 | 477.1397 | -2.04 | 167221 | -H | C_23_H_26_O_11_ | | M/Z315.1061 [M-H-GIc]^-^ | | Persicoside | | SMJ |
| 91 | 7.65 | 623.16 | 623.1612 | -1.74 | 6394 | -H | C_28_H_32_O_16_ | | M/Z477.1357 [M-H-GIc]^-^M/Z355.1061 [M-H-2GIc]^-^ | | Isorhamnetin-3-O-β-D-Rutinoside | | SHZ |
| 92 | 7.65 | 625.1727 | 625.1759 | -0.4 | 798 | +H | C_28_H_32_O_16_ | | M/Z 317.0627[M+H-C_12_H_20_O_9_]^+^ | | Isorhamnetin-3-O-β-D-Rutinoside | | SMJ |
| 93 | 7.67 | 639.1887 | 639.1925 | -4.43 | 90230 | -H | C_29_H_36_O_16_ | | M/Z 477.1578 [M-H-C_6_H_6_O_6_]^-^ | | Plantamajoside | | SMJ |
| 94 | 7.88 | 547.1168 | - | - | 10600 | -H | - | | - | | Unknown | | SMJ |
| 95 | 7.9 | 653.1688 | 653.1717 | 1.11 | 996 | +H | C_29_H_32_O_17_ | | M/Z 287.0576 [M+H-C_14_H_22_O_9_]^+^、M/Z 163.0381 [M+H-C_6_H_4_O_3_]^+^ | | Isoscutellarein 7-O-2)glucopyranoside^b^ | | SMJ |
| 96 | 8.01 | 269.1902 | - | - | 2459 | +H | - | | - | | Unknown | | SHZ |
| 97 | 8.02 | 529.2595 | 529.259 | -0.47 | 7650 | -H | C_26_H_42_O_11_ | | M/Z451.1312[M-H-CH_2_O_4_]^-^ | | Pharboside D | | SHZ |
| 98 | 8.05 | 653.1688 | 653.1717 | 1.13 | 1780 | +H | C_29_H_32_O_17_ | | M/Z 287.0576 [M+H-C_14_H_22_O_9_]^+^、M/Z 163.0381 [M+H-C_6_H_4_O_3_]^+^ | | 2''-O-Acetylrutin^b^ | | SMJ |
| 99 | 8.13 | 515.1179 | 515.119 | -1.63 | 1253956 | -H | C_25_H_24_O_12_ | | M/Z353.0852[M-H-C_9_H_6_O_3_]^-^  M/Z191.0549 [M-H-2C_9_H_6_O_3_]^-^ | | 1,5  Dicaffeoylquinic acids^a^ | | SHZ |
| 100 | 8.13 | 517.1329 | 517.1309 | -3.20 | 15266 | +H | C_25_H_24_O_12_ | | M/Z355.1023[M+H-C_9_H_6_O_3_]^+^  M/Z193.0339 [M+H-2C_9_H_6_O_3_]^+^ | | 1,5 dicaffeoyl quinic acid | | SHZ |
| 101 | 8.15 | 149.0970 | 149.0971 | 0.98 | 1099 | +H | C_10_H_12_O | | M/Z121.0999 [M+H-CO]^+^ | | Estragole | | SHZ |
| 102 | 8.44 | 679.5096 | - | - | 5260 | - | - | | - | | Unknown | | SMJ |
| 103 | 8.49 | 835.1628 | - | - | 44200 | 2M-H | - | | - | | Unknown | | SHZ |
| 104 | 8.61 | 445.0794 | 445.0771 | 1.84 | 3637 | -H | C_21_H_18_O_11_ | | M/Z393.0850 [M-H-CH_8_O_2_]^-^ | | Apigenin 7-glucuronide | | SMJ |
| 105 | 8.62 | 447.0916 | 447.0932 | 1.00 | 875 | +H | C_21_H_18_O_11_ | | M/Z 365.1220 [M+H-C_4_H_2_O_2_]^+^ | | Apigenin 7-glucuronide | | SMJ |
| 106 | 8.81 | 163.0426 | - | - | 667 | +H | - | | - | | Unknown | | SMJ |
| 107 | 8.82 | 559.1169 | - | - | 3040 | -H | - | | - | | Unknown | | SMJ |
| 108 | 9.04 | 491.157 | 491.1554 | 1.13 | 4018 | -H | C_24_H_28_O_11_ | | M/Z423.1646 [M-H-C_9_H_6_O_3_]^-^ | | Picroside I | | SMJ |
| 109 | 9.12 | 183.0667 | 183.066 | 0.80 | 498 | +H | C_9_H_10_O_4_ | | M/Z 165.0535 [M+H-H_2_O]^+^ | | Ethyl 3,4-dihydroxybenzoate | | SHZ |
| 110 | 9.33 | 385.1525 | - | - | 1710 | -H | - | | - | | Unknown | | SMJ |
| 111 | 9.59 | 607.1425 | - | - | 2660 | -H | - | | - | | Unknown | | SMJ |
| 112 | 9.96 | 813.276 | 813.2817 | 3.54 | 6280 | -H | C_37_H_50_O_20_ | | M/Z651.2294[M-H-C_6_H_10_O_5_]^-^ | | Jionoside B^b^ | | SHZ |
| 113 | 10.21 | 177.0539 | - | - | 281 | +H | - | | - | | Unknown | | SHZ |
| 114 | 10.22 | 431.0972 | 431.0978 | 0.69 | 4230 | -H | C_21_H_20_O_10_ | | - | | Apigenin-7-O-glucoside^b^ | | SHZ |
| 115 | 10.23 | 651.2256 | 651.2289 | -3.84 | 19637 | -H | C_31_H_40_O_15_ | | - | | Martynoside | | SMJ |
| 116 | 10.46 | 277.1782 | - | - | 381 | +H | - | | - | | Unknown | | SHZ |
| 117 | 10.58 | 505.1639 | 505.1651 | 1.18 | 2620 | -H | C_32_H_26_O_6_ | | - | | 2,2'-dihydroxy-4,4',7,7'-Tetramethoxy-1,1'-biphenanthrene^b^ | | SMJ |
| 118 | 10.9 | 401.0844 | 401.0873 | 3.61 | 5100 | -H | C_20_H_18_O_9_ | | M/Z239.0567[M-H-C_8_H_2_O_4_]^-^ | | 7^-^ Carboxymethoxyloxy-3',4',5-trimethoxyflavone^b^ | | SHZ |
| 119 | 10.92 | 187.0748 | 187.0759 | 2.94 | 233 | +H | C_12_H_10_O_2_ | | M/Z 165.0535 [M+H-C_4_H_5_O_3_]^+^ | | Chimaphylin | | SHZ |
| 120 | 11 | 483.1842 | 483.1808 | -0.35 | 8760 | -H | C_30_H_28_O_6_ | | - | | Shancilin^b^ | | SHZ |
| 121 | 11.06 | 381.1173 | 381.1186 | 1.74 | 172413 | -H | C_18_H_22_O_9_ | | M/Z 179.0332[M-H-C_6_H_6_O_6_]^-^ | | Ningposide B^b^ | | SMJ |
| 122 | 11.07 | 163.0381 | - | - | 622 | +H | - | | - | | Unknown | | SHZ |
| 123 | 11.23 | 331.1513 | - | - | 3930 | -H | - | | - | | Unknown | | SHZ |
| 124 | 11.23 | 297.1525 | 297.1488 | 0.30 | 2593 | +H | C_19_H_20_O_3_ | | M/Z 279.0920 [M+H–H_2_O]^+^、M/Z 264.1260 [[M+H-H_2_O-CH_3_]^+^、M/Z 251.1654[M+H-H_2_O-CO]^+^ | | Cryptotanshinone | | SHZ |
| 125 | 11.36 | 469.3337 | 469.3313 | 0.10 | 1693 | +H | C_30_H_44_O_4_ | | M/Z 453.1260 [M+H-C_14_H_22_O_9_]^+^ | | Aquaticol | | SMJ |
| 126 | 11.42 | 271.0588 | 271.0611 | 1.00 | 2448 | +H | C_15_H_10_O_5_ | | M/Z177.0585[M+H-C_5_H_2_O_2_]^+^  M/Z163.0426[M+H-C_5_H_2_O_2_-CH_2_]^+^  M/Z79.0605[M+H-C_5_H_2_O_2_-CH_2_-C_3_H_3_O_3_]^+^ | | Apigenin | | SMJ |
| 127 | 11.62 | 275.16 | 275.1647 | 8.54 | 4080 | +H | C_17_H_22_O_3_ | | - | | Nervogenic acid^b^ | | SHZ |
| 128 | 11.65 | 329.2317 | 329.2325 | 1.21 | 43129 | -H | C_18_H_34_O_5_ | | M/Z229.1436[M-H-C_5_H_8_O_2_]^-^ | | 9,12,13-Trihydroxy-10-octadecenoic acid^b^ | | SHZ |
| 129 | 11.86 | 287.2229 | 242.2246 | 2.95 | 165517 | -H | C_15_H_30_O_2_ | | M/Z141.1313[M-H-C_5_H_8_O_2_]^-^ | | Glycerides C14-18^b^ | | SMJ |
| 130 | 12.03 | 180.102 | 180.1024 | 1.11 | 4270 | +H | C_10_H_13_NO_2_ | | M/Z 137.0492 [M+H-CO_2_]^+^ | | N-methyl phenylalanine^b^ | | SHZ |
| 131 | 12.23 | 315.1596 | 270.162 | 3.84 | 148524 | +HCOO | C_18_H_22_O_2_ | | M/Z253.1587[M-H-O]^-^ | | Estrone^b^ | | SHZ |
| 132 | 12.50 | 315.1561 | - | - | 7214 | -H | - | | - | | Unknown | | SHZ |
| 133 | 12.6 | 316.286 | 316.2851 | -1.42 | 386 | +H | C_18_H_37_NO_3_ | | - | | Myristic acid diethanolamide^b^ | | SHZ |
| 134 | 12.9 | 265.1473 | - | - | 11700 | -H | - | | - | | Unknown | | SHZ |
| 135 | 12.98 | 194.1181 | 194.1162 | 4.89 | 5970 | +H | C_11_H_15_NO_2_ | | M/Z134.0615[M+H-C_2_H_4_O_2_]^+^ | | Salsoline^b^ | | SHZ |
| 136 | 13.05 | 368.9751 | - | - | 15100 | -H | - | | - | | Unknown | | SHZ |
| 137 | 13.12 | 313.2336 | - | - | 52900 | -H | - | | - | | Unknown | | SHZ |

**Note:** **a:** has been compared with the reference standard; **b:** Discovered for the first time from anti swelling and pain relieving patche

**Table S7** Identification results of transdermal components detected by UHPLC-QTOF-MS/MS in the positive and negative ion mode of DAP

| NO. | TR (min) | Observed  M/Z | Theoretical  M/Z | Mass  error | Response | Adducts | Formulas | Fragmentation patterns | Potential compounds | Herbs |  |  |
| --- | --- | --- | --- | --- | --- | --- | --- | --- | --- | --- | --- | --- |
| 1 | 0.84 | 195.0515 | 150.0529 | 0.25 | 101000 | +HCOO | C_5_H_10_O_5_ | - | DL-Arabinose | SHZ |  |  |
| 2 | 0.86 | 341.1095 | 341.1086 | 0.27 | 31700 | -H | C_12_H_22_O_11_ | M/Z195.0522[M-H-C_6_H_5_O_5_]^-^  M/Z 179.0568[M-H-C_6_H_6_O_6_]^-^ | Sucrose | SHZ |  |  |
| 3 | 0.86 | 381.0799 | 381.0821 | 3.10 | 48700 | +H | C_17_H_16_O_10_ | M/Z 365.1018 [M+H-O]^+^ | 8-O-beta-D-Glucopyranosyl-5-hydroxypsoralen | SHZ |  |  |
| 7 | 1.22 | 191.0209 | 191.0192 | -4.18 | 70300 | -H | C_6_H_8_O_7_ | M/Z173.0133[M-H-H_2_O]^-^M/Z129.0203[M-H-H_2_O^-^C_2_O]^-^M/Z111.0098[M-H^-^H_2_O-C_2_O-H_2_O]^-^ | Citric acid | SMJ |  |  |
| 11 | 1.45 | 123.0467 | 123.0446 | 1.27 | 1163 | +H | C_7_H_6_O_2_ | M/Z119.0517[M+H+CH_2_-O_2_]^+^ | 4-Hydroxybenzaldehyde | SHZ |  |  |
| 18 | 1.95 | 283.0665 | 283.0679 | 2.47 | 74600 | -H | C_10_H_12_N_4_O_6_ | M/Z151.0252[M-H-C5H8O4]^-^ | Xanthosine | SHZ |  |  |
| 27 | 2.94 | 255.0503 | - | - | 12500 | -H | - | - | Unknown | SHZ |  |  |
| 29 | 3.34 | 153.0180 | 153.0188 | 0.64 | 48100 | -H | C_7_H_6_O_4_ | M/Z109.03[M-H-CO_2_]^-^ | Protocatechuic  acid | SMJ |  |  |
| 32 | 3.56 | 353.0852 | 353.0873 | 2.97 | 1560 | -H | C_16_H_17_O_9_ | M/Z 191.0549[M-H-C_9_H_6_O_3_]^-^ | 4-Caffeoylquinic acid | SHZ |  |  |
| 33 | 3.81 | 188.0759 | 188.0711 | -4.55 | 20300 | +H | C_11_H_9_NO_2_ | M/Z146.0608[M+H-CHO_2_]^+^  M/Z118.0667[M+H-CHO_2_-C_2_H_4_]^+^ | 3-Indoleacrylic acid | SHZ |  |  |
| 35 | 3.89 | 373.1127 | 373.1135 | -0.47 | 1420 | -H | C_16_H_22_O_10_ | M/Z193.0508[M-H-C_9_H_8_O_4_]^-^M/Z149.0583[M-H-C_9_H_8_O_4_-C_2_H_4_O]^-^ | Geniposidic  acid | SMJ |  |  |
| 36 | 3.72 | 637.1796 | 637.1769 | -2.11 | 10400 | -H | C_29_H_34_O_16_ | M/Z 499.1431 [M-H-C_6_HO_4_]^-^ | Ombuoside | SHZ |  |  |
| 40 | 4.55 | 451.1312 | - | - | 1340 | -H | - | - | Unknown | SHZ |  |  |
| 41 | 4.53 | 291.0978 | 291.1021 | 7.99 | 1980 | +H | C_19_H_14_O_3_ | M/Z172.0775 [M+H-C_4_H_7_O_4_]^+^ | Aurin | SHZ |  |  |
| 44 | 4.63 | 583.1749 | 538.1686 | 8.15 | 3512 | +HCOO | C_25_H_30_O_13_ | M/Z 375.0742 [M-H-C_6_H_10_O_5_]^-^ | 6-Feruloylcatalpol | SMJ |  |  |
| 45 | 4.75 | 707.1803 | 707.1824 | 1.48 | 55000 | 2M-H | C_32_H_36_O_18_ | M/Z191.0549[M-H-C_9_H_6_O_3_]-  M/Z179.0549[M-H-C_7_H_10_O_5_]^-^  M/Z135.0549[M-H-C_8_H_10_O_5_]^-^ | Chlorogenic acid | SHZ |  |  |
| 46 | 4.70 | 163.0389 | 163.0395 | -4.29 | 23007 | +H | C_9_H_6_O_3_ | M/Z 135.0455 [M+H-C_2_H_4_]^+^ | 7-Hydroxycoumarin | SHZ |  |  |
| 47 | 5.05 | 239.0567 | 194.0579 | 2.56 | 1345 | +HCOO | C_10_H_10_O_4_ | M/Z179.0332[M-H-CO_2_]- | Ferulate | SMJ |  |  |
| 51 | 5.57 | 179.0332 | 179.0345 | 3.63 | 24100 | -H | C_9_H_8_O_4_ | M/Z135.0447[M-H-CO_2_]^-^ | Caffeic acid | SHZ |  |  |
| 62 | 6.47 | 497.1286 | 497.1295 | -1.53 | 59500 | -H | C_22_H_26_O_13_ | M/Z335.0782[M-H-C_6_H_10_O_5_]^-^M/Z221.0449[M-H-C_6_H_10_O_5_-C_4_H_2_O_4_]^-^、M/Z153.0180[M-H-C_6_H_10_O_5_-C_9_HO_4_]^-^M/Z109.0300[M-H-C_6_H_10_O_5_-C_9_HO_4_-CO_2_]^-^ | Verproside | SMJ |  |  |
| 65 | 6.48 | 627.1546 | 627.1561 | -1.27 | 25700 | +H | C_27_H_30_O_17_ | M/Z303.0492 [M+H-C_12_H_20_O_10_]^+^ | Quercetin-3-sophoroside | SMJ |  |  |
| 66 | 6.50 | 625.1393 | 625.1405 | 0.88 | 17124 | -H | C_27_H_30_O_17_ | M/Z301.0366[M-H-2GIc]^-^ | Quercetin-3-sophoroside | SMJ |  |  |
| 75 | 7.44 | 609.1454 | 609.1456 | 0.14 | 863 | -H | C_27_H_30_O_16_ | M/Z285.0428[M-H-C_12_H_20_O_10_]^-^M/Z193.0508[M-H-C_12_H_20_O_10_-C_6_H_4_O]- | Rutin | SMJ |  |  |
| 77 | 7.77 | 481.1353 | 481.1346 | -0.76 | 30100 | -H | C_22_H_26_O_12_ | M/Z319.0816[M-H-C_6_H_10_O_5_]^-^ | Catalposide | SMJ |  |  |
| 83 | 8.08 | 511.1442 | 511.1452 | -1.42 | 46200 | -H | C_23_H_28_O_13_ | M/Z349.0933[M-H-GIc]^-^M/Z235.0609[M-H-GIc-C_5_H_6_O_3_]^-^ | Picroside II | SMJ |  |  |
| 85 | | 8.10 | 523.1444 | 523.1452 | -1.31 | 12200 | -H | C_24_H_28_O_13_ | M/Z349.0933[M-H-GIc]  M/Z235.0609[M-H- GIc -C_5_H_6_O_3_]^-^  Z167.0332.[M-H-GIc- C_5_H_6_O_3_-CO-C_7_O_4_H_5_]^-^、M/Z123.0473[M-H-GIc- C_5_H_6_O_3_-CO-CO_2_]^-^ | Verminoside | SMJ |  |
|  |  |  |  |  |  |  |  |  |  |  |  |  |
| 93 | | | 8.81 | 639.1887 | 639.1925 | -4.43 | 2231 | -H | C_29_H_36_O_16_ | M/Z 477.1578 [M-H-C_6_H_6_O_6_]^-^ | Plantamajoside | SMJ |
| 94 | | | 8.88 | 547.1168 | - |  | 1160 | -H |  | - | Unknown | SMJ |
| 99 | | | 9.00 | 515.1179 | 515.119 | -1.66 | 184000 | -H | C_25_H_24_O_12_ | M/Z353.0852[M-H-C_9_H_6_O_3_]^-^  M/Z191.0549 [M-H-2C_9_H_6_O_3_]^-^ | 1,5 Dicaffeoylquinic acids^a^ | SHZ |
| 100 | | | 8.97 | 517.1342 | 517.1309 | -3.22 | 31005 | +H | C_25_H_24_O_12_ | M/Z355.1023[M+H-C_9_H_6_O_3_]^+^  M/Z193.0339 [M+H-2C_9_H_6_O_3_]^+^ | 1,5 dicaffeoyl quinic acid^a^ | SHZ |
| 103 | | | 9.36 | 835.1632 | - | - | 11000 | 2M-H | - | - | Unknown | SHZ |
| 108 | | | 9.64 | 491.1573 | 491.1554 | 1.23 | 3240 | -H | C_24_H_28_O_11_ | M/Z423.1646 [M-H-C_9_H_6_O_3_]^-^ | Picroside I | SMJ |
| 114 | | | 11.12 | 431.0977 | 431.0978 | 0.99 | 1834 | -H | C_21_H_20_O_10_ | - | Apigenin-7-O-glucoside | SHZ |
| 115 | | | 11.25 | 651.2266 | 651.2289 | -3.74 | 3580 | -H | C_31_H_40_O_15_ | - | Martynoside | SMJ |
| 117 | | | 11.62 | 505.1643 | 505.1651 | 1.58 | 2620 | -H | C_32_H_26_O_6_ | - | 2,2'-dihydroxy-4,4',7,7'-Tetramethoxy-1,1'-biphenanthrene | SMJ |
|  |  |  |  |  |  |  |  |  |  |  |  |  |
|  |  |  |  |  |  |  |  |  |  |  |  |  |
|  |  |  |  |  |  |  |  |  |  |  |  |  |
|  |  |  |  |  |  |  |  |  |  |  |  |  |
|  |  |  |  |  |  |  |  |  |  |  |  |  |
|  |  |  |  |  |  |  |  |  |  |  |  |  |
|  |  |  |  |  |  |  |  |  |  |  |  |  |
|  |  |  |  |  |  |  |  |  |  |  |  |  |
|  |  |  |  |  |  |  |  |  |  |  |  |  |
