# Supplementary material for: Detumescence Analgesic Plaster mitigates knee osteoarthritis via active ingredients targeting mitochondrial complex 1/AMPK/MYL3-regulated cartilage homeostasis
Source: Chin Med. 2025 Oct 20;20:175. doi: 10.1186/s13020-025-01215-w (PMC12536539; doi:10.1186/s13020-025-01215-w)
Supplement: Supplementary file 3 — Additional file 3: Fig. S1 A 206 reverse regulatory genes of DAP were screened and B-D GO enrichment analysis of the gene of anti-KOA effect of DAP. Fig. S2. Interaction between MYL3 protein and p-AMPK protein. A ZDOCK software predicts the interaction between AMPK and MYL3 proteins. (Purple area represents AMPK protein, blue area represents MYL3 protein, white circled area represents predicted binding region, yellow represents hydrogen bond. B-D Western blot was used to detect endogenous interaction between AMPK and MYL3 protein (n =3). Fig. S3. BPI chromatogram of DAP compounds. (A: negative ion mode; B: positive ion mode). Fig. S4. BPI chromatogram of DAP transdermal component negative ion mode. (A: Blank skin component; B: Transdermal component). Fig. S5. BPI chromatogram of DAP transdermal component positive ion mode. (A Blank skin component; B Transdermal component.) Fig. S6.CCK-8 was used to detect the changes in cell proliferation after 24 h treated with 19 compounds at different concentrations (0, 25, 50, 100 μM). Fig. S7. Top 60% of transdermal compounds with anti-inflammatory activities (n =6). [file 13020_2025_1215_MOESM3_ESM.docx]

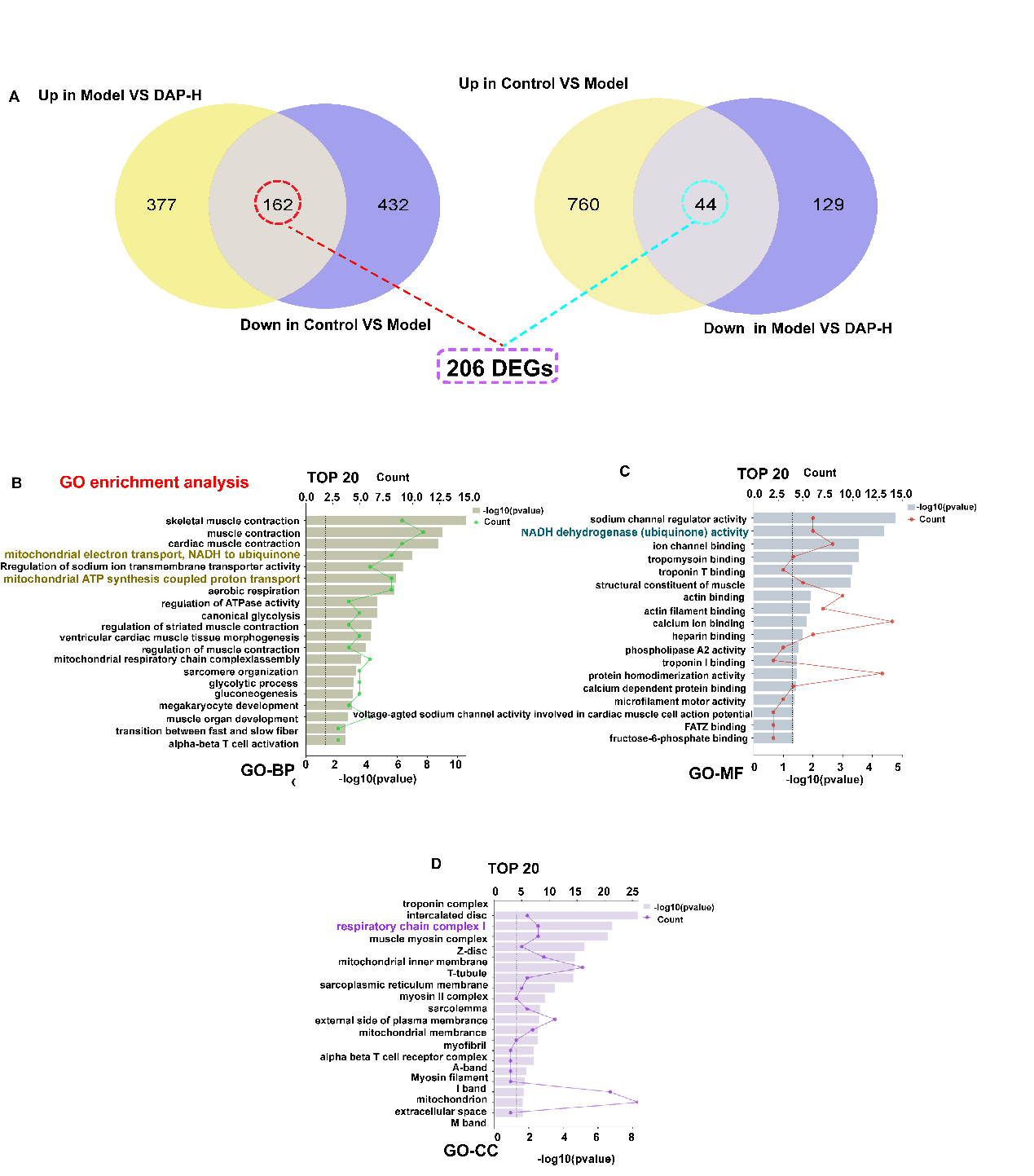
**Supplementary Fig S1-S7**

**Fig S1.** (A) 206 reverse regulatory genes of DAP were screened and (B-D) GO enrichment analysis of the gene of anti-KOA effect of DAP.


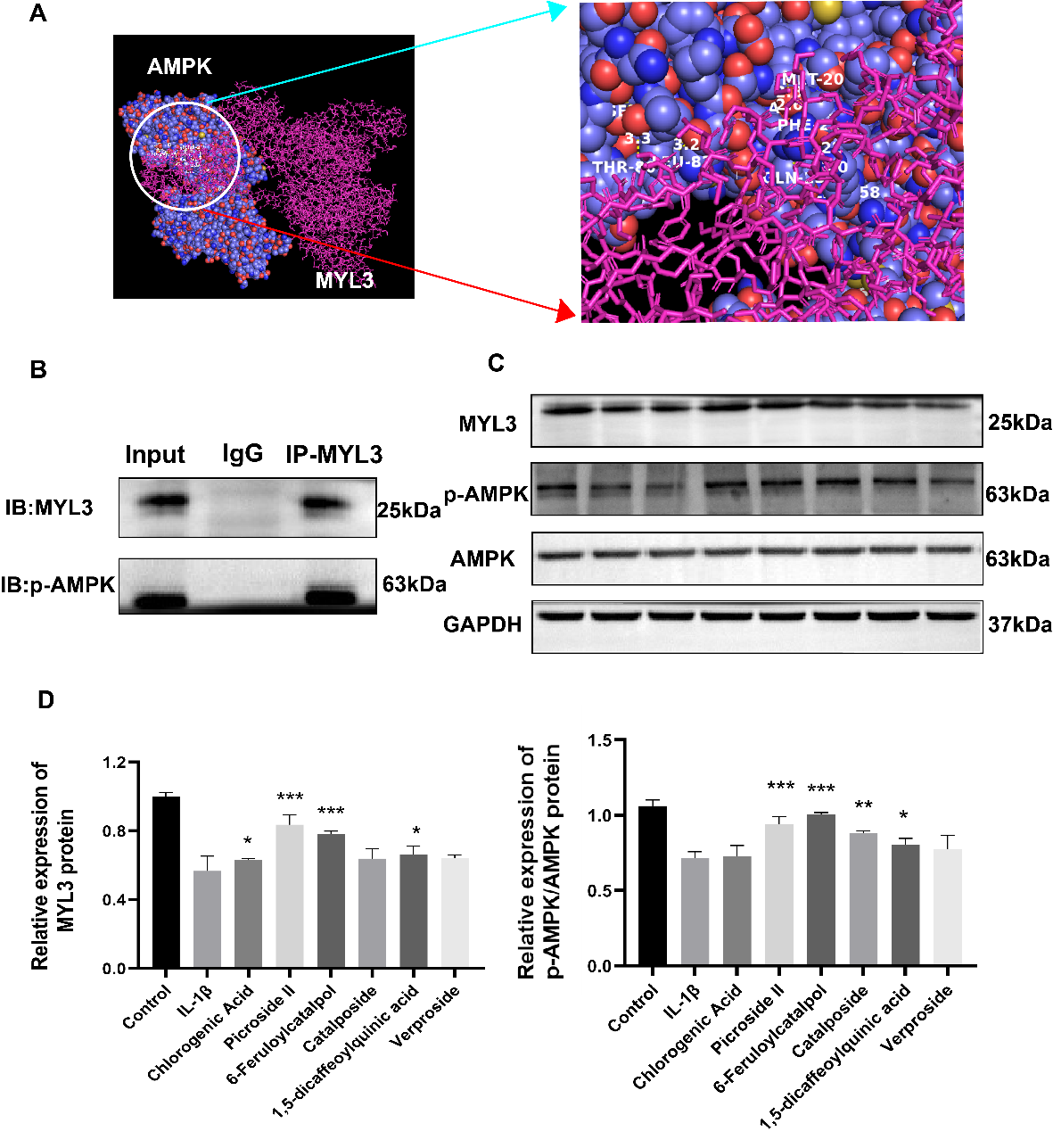


**Fig S2.** Interaction between MYL3 protein and p-AMPK protein. (A) ZDOCK predicted structure of AMPK MYL3 complex: purple area represents AMPK protein, blue area represents MYL3 protein, white circled area represents predicted binding region, yellow represents hydrogen bond, (B-D) Western blot was used to detect endogenous interaction between AMPK and MYL3 protein (n =3).


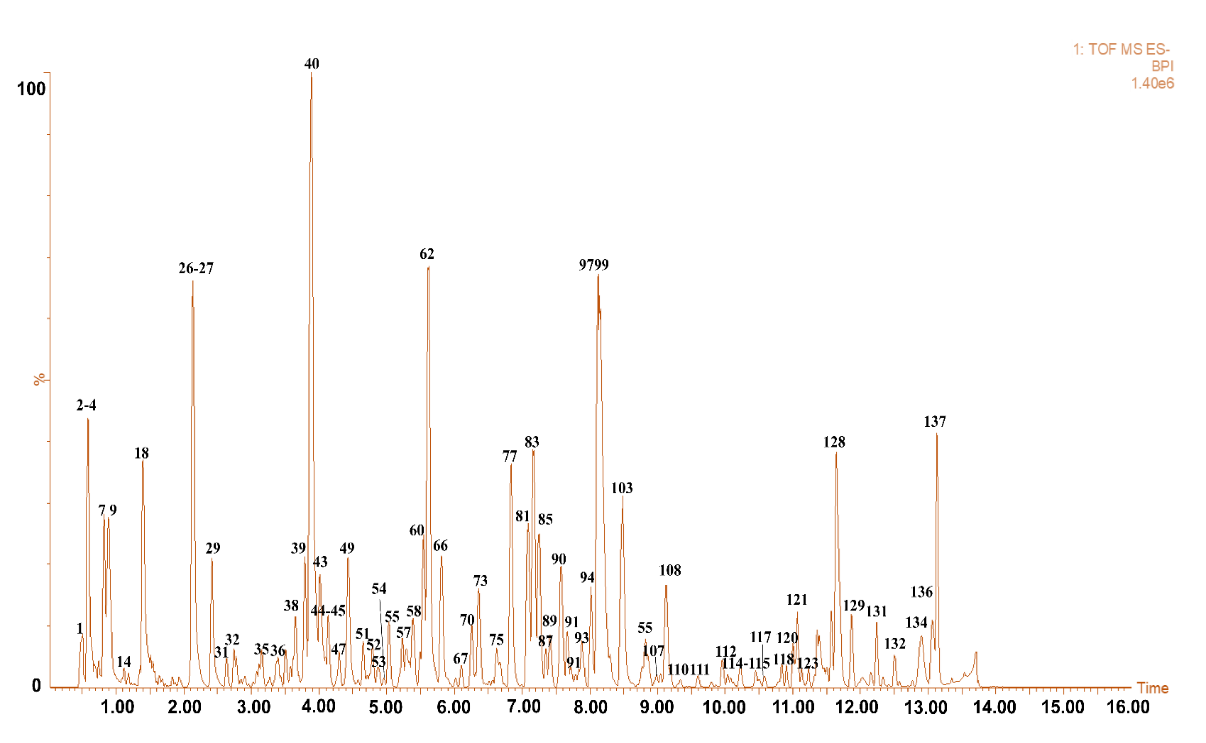

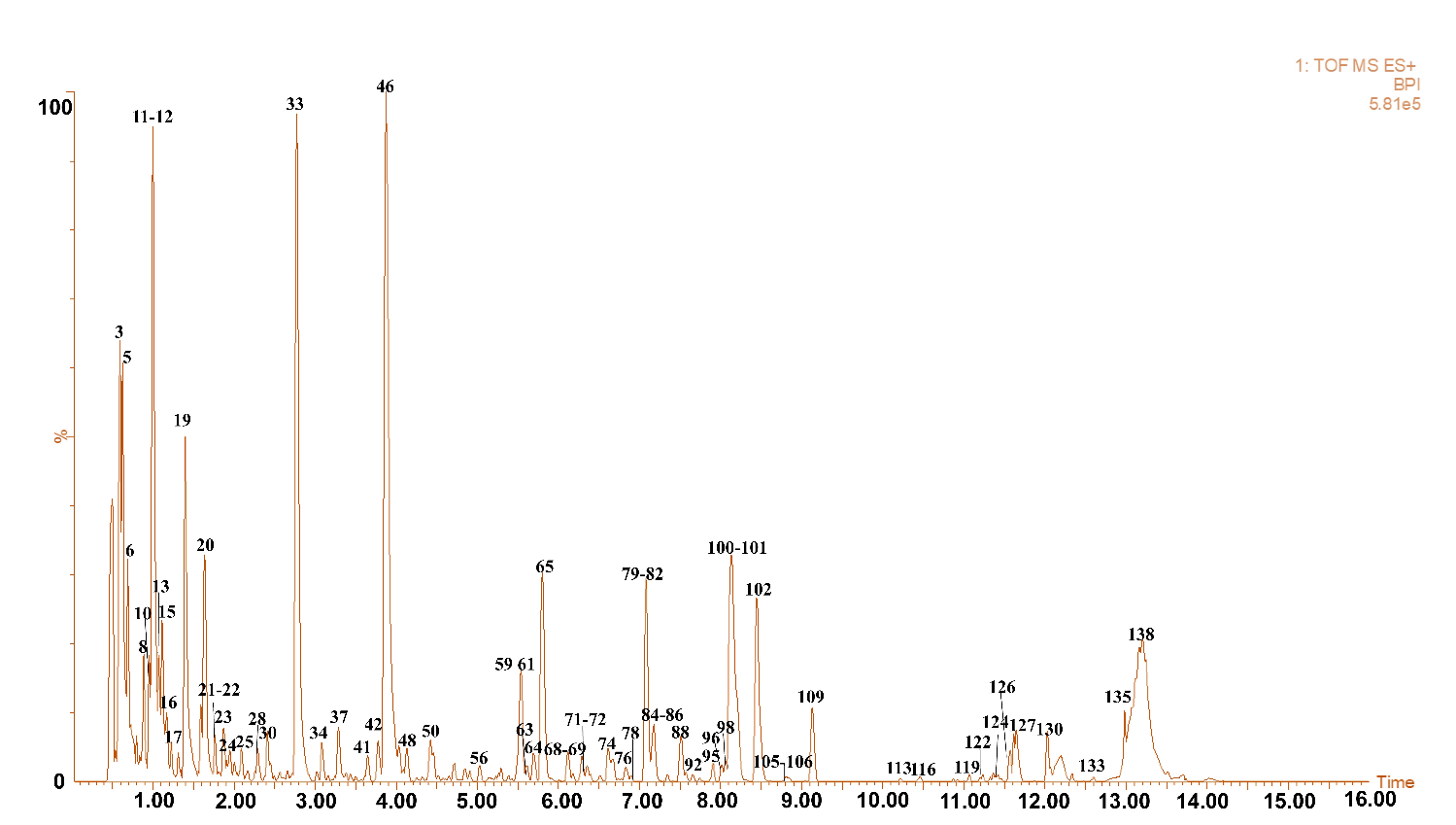


**Fig S3.** Base peak chromatogram (BPI) chromatogram of DAP compound UPLC-QTOF-MS/MS. (A: negative ion scan B: positive ion scan).


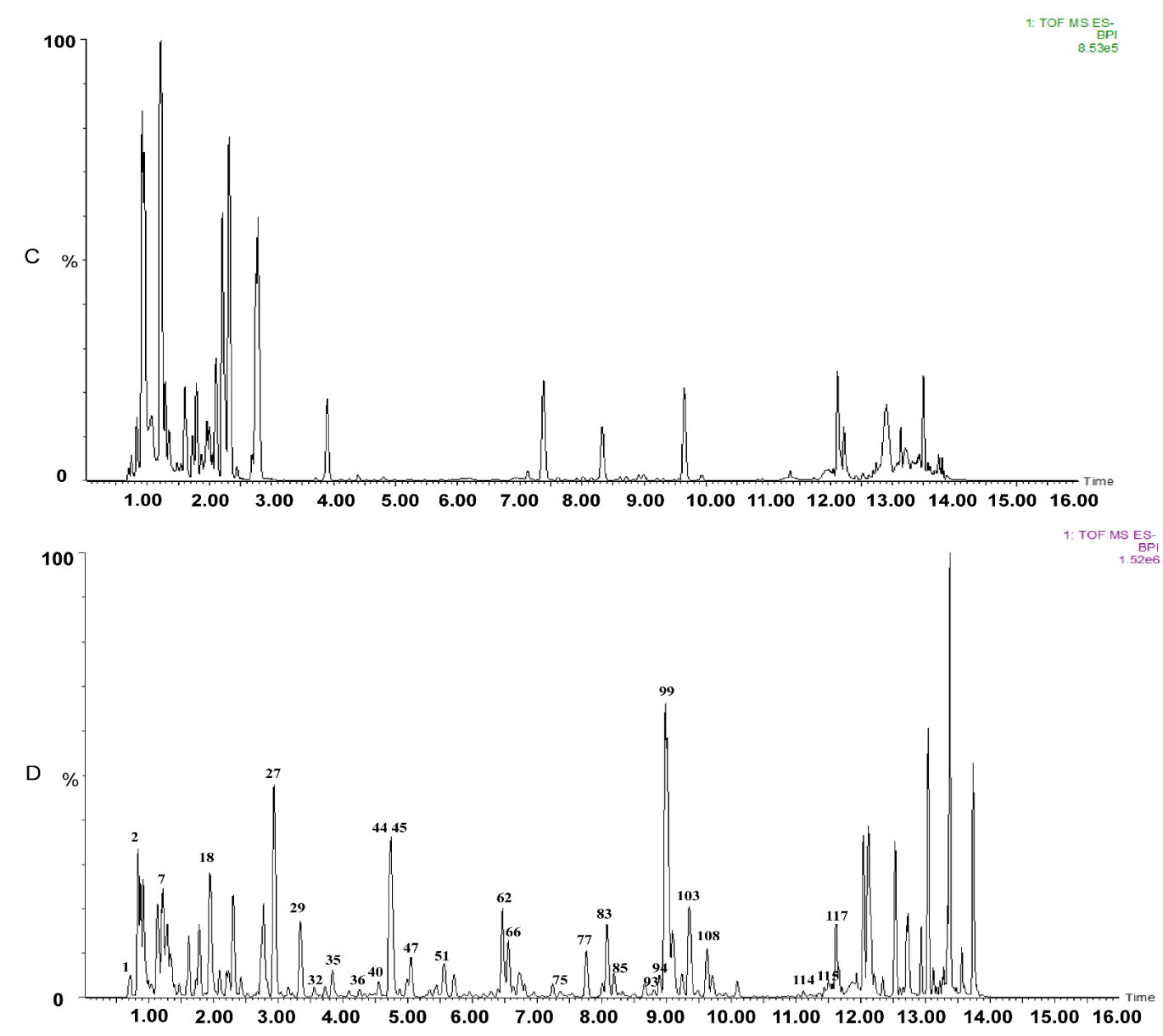


**Fig S4.** BPI chromatogram of UPLC-QTOF-MS/MS in DAP transdermal component negative ion mode. (A: blank skin component B: transdermal component)


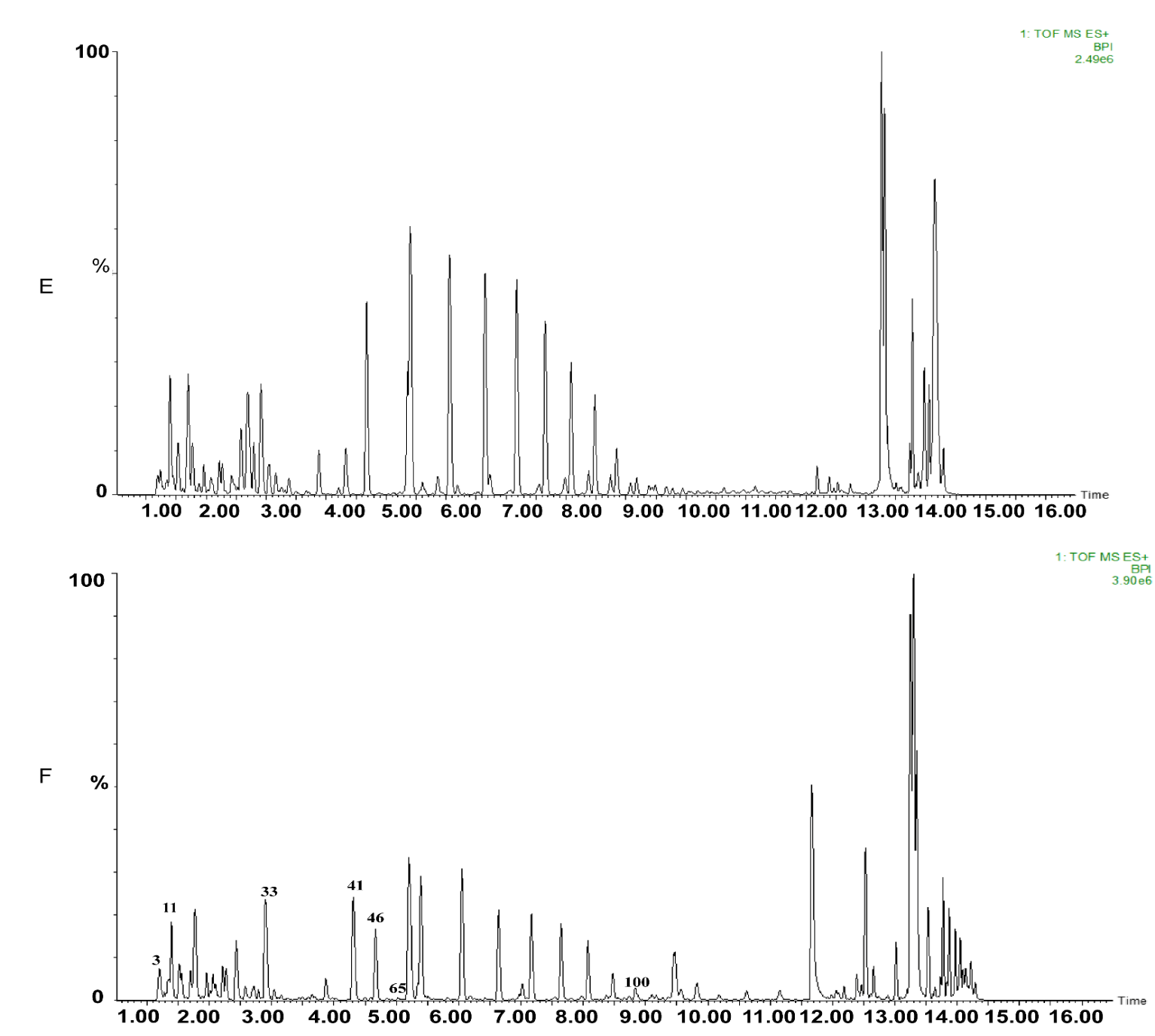


**Fig S5.** BPI chromatogram of UPLC-QTOF-MS/MS in DAP transdermal component positive ion mode A: blank skin component B: transdermal component.


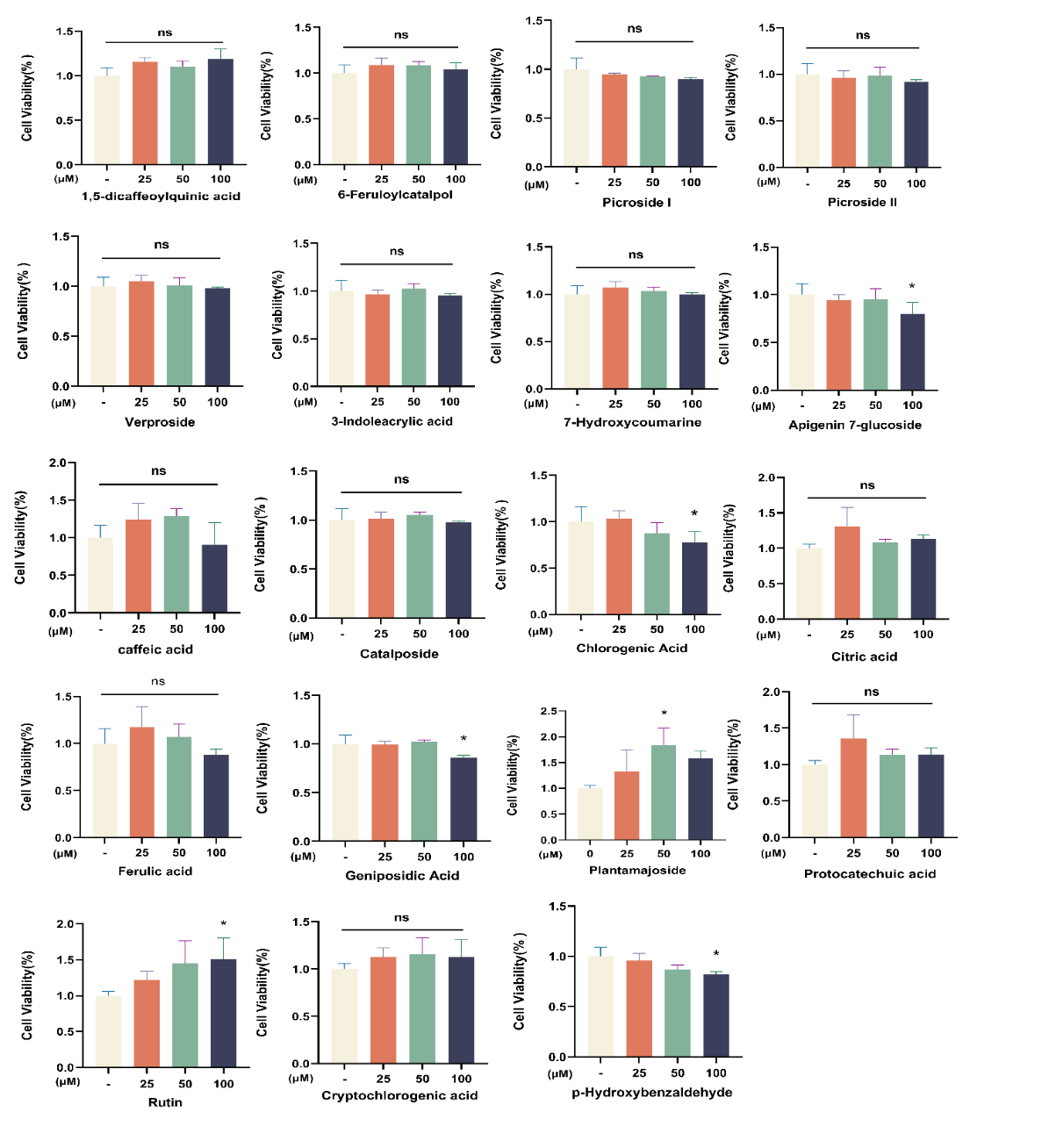


**Fig S6.**CCK-8 was used to detect the changes in cell proliferation after 24 h of treatment with 19 compounds at different concentrations (0, 25, 50, 100 μM).


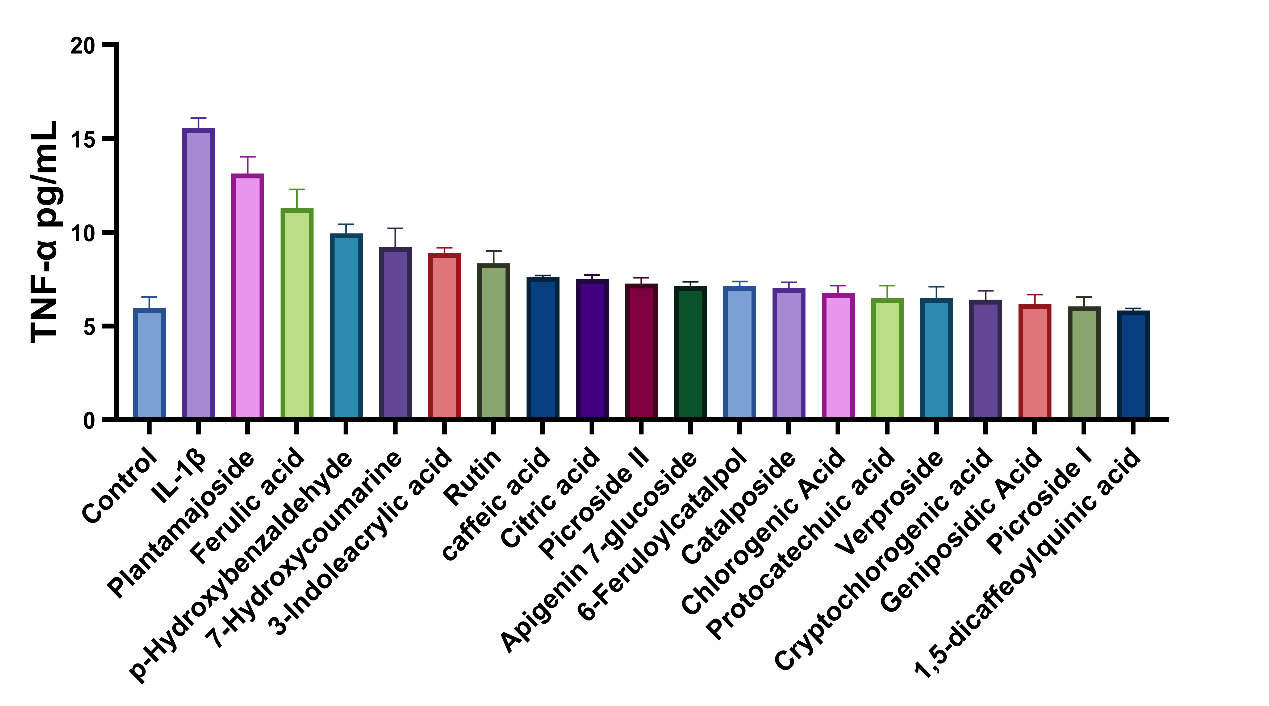


**Fig S7.** Top 60% of transdermal compounds with anti-inflammatory activity (n =5)
